# The Role of Arg13 in Protein Phosphatase M tPphA from *Thermosynechococcus elongatus*

**DOI:** 10.1155/2012/272706

**Published:** 2012-06-06

**Authors:** Jiyong Su, Karl Forchhammer

**Affiliations:** Interfaculty Institute for Microbiology and Infection Medicine, Department of Organismic Interactions, University of Tübingen, 72076 Tübingen, Germany

## Abstract

A highly conserved arginine residue is close to the catalytic center of PPM/PP2C-type protein phosphatases. Different crystal structures of PPM/PP2C homologues revealed that the guanidinium side chain of this arginine residue can adopt variable conformations and may bind ligands, suggesting an important role of this residue during catalysis. In this paper, we randomly mutated Arginine 13 of tPphA, a PPM/PP2C-type phosphatase from *Thermosynechococcus elongatus*, and obtained 18 different amino acid variants. The generated variants were tested towards *p*-nitrophenyl phosphate and various phosphopeptides. Towards *p*-nitrophenyl phosphate as substrate, twelve variants showed 3–7 times higher *K*_*m*_ values than wild-type tPphA and four variants (R13D, R13F, R13L, and R13W) completely lost activity. Strikingly, these variants were still able to dephosphorylate phosphopeptides, although with strongly reduced activity. The specific inability of some Arg-13 variants to hydrolyze *p*-nitrophenyl phosphate highlights the importance of additional substrate interactions apart from the substrate phosphate for catalysis. The properties of the R13 variants indicate that this residue assists in substrate binding.

## 1. Introduction

The protein serine/threonine phosphatases constitute two large families, the phosphoprotein phosphatases (PPP) and the metal-dependent protein phosphatases (PPM) and one small family, the aspartate-based phosphatases [1]. The human PPM member PP2C*α* [2] has been the defining representative of the PPM family, which is therefore also referred as the PP2C family. PP2C phosphatases are widely present in eukaryotes and prokaryotes where they regulate diverse signaling pathways involved in central cellular processes, such as cell proliferation, stress responses, or metabolic activity [3]. Recently, several crystal structures of bacterial and plant PP2Cs were solved, and they all show that five highly conserved aspartate residues constitute a negative charged pocket that coordinates three Mg^2+^/Mn^2+^ (M1, M2, and M3) ions in the catalytic center [4–9]. All three metal ions were proven by mutational analysis of the coordinating Asp residues to be essential for the activity of PP2Cs [2, 10–12]. Recently, we have reported that the third metal (M3) in the catalytic centre of tPphA (a PP2C member from *Thermosynechococcus elongatus*) takes part in catalysis, presumably by activating a water molecule to act as proton donor for the leaving group [11]. Recently, further regions in the periphery of the catalytic core of tPphA were identified to play roles in substrate recognition: His-39, a conserved residue in bacterial PP2C members, and the variable flap-subdomain facing M3 [13].

According to the PP2C motif nomenclature of Bork et al. 1996 [14], a conserved arginine residue located in the beginning of motif 1 and structurally close to the catalytic center of PP2C may play an important role in catalysis as deduced from structural analysis. The guanidinium side chain of this arginine residue could adopt different conformations in various PP2C structures; the crystal structures of human PP2C*α* [2] and MspP (a PP2C member from *Mycobacterium smegmatis*) [6] in complex with a phosphate ion revealed that this arginine residue is hydrogen bonded with one oxygen atom of the phosphate ion in the catalytic pocket. Enzymatic assays of a human PP2C*α* variant, where the Arg-33 residue was replaced by Ala showed that this arginine residue is important for binding *p*-nitrophenyl phosphate (*p*NPP), since the variant R33A shows 8-fold higher *K*_*m*_ than wild-type phosphatase [12]. The crystal structures of MspP in complex with sulphate and cacodylate were also solved [6]. In the crystal structure of MspP with sulphate, the conserved arginine residue (Arg-17 of MspP) is hydrogen bonded with the sulphate ion, which is dislodged from the catalytic center of MspP. It was, therefore, suggested that this conformation of Arg-17 adapt to the incoming phosphoprotein substrate or the outgoing inorganic phosphate after catalysis, implying that Arg-17 might assist in releasing inorganic phosphate after catalysis [6]. In the crystal structure of MspP with cacodylate, MspP Arg-17 makes a hydrogen bond with a water molecule, whereas the two oxygen atoms of cacodylate are coordinated by M1 and M2. A crystal of *Sa*STP (a PP2C member from *Streptococcus agalactiae*) with four monomers (A, B, C, and D) in the asymmetric unit revealed monomer C in a conformation, which interacted with the flap subdomain of the adjacent monomer A [7]. The corresponding arginine residue (Arg-13 of *Sa*STP) side chain showed two different conformations in monomer A and C, respectively. In monomer A, its conformation is similar to human PP2C*α*, where it binds phosphate [2]. In monomers C, Arg-13 adopts a new conformation, resembling Arg-17 of MspP in complex with a sulphate ion [6]; the side chain is rotated so that the guanidine group points away from the active site and binds a serine residue (Ser155) of the monomer A. A further indication for a role of this arginine residue during catalysis comes for the crystal structure of human PP2C*α*, whose metals had been removed [2]. Refinement of the metal-free human PP2C*α* structure revealed that it was almost identical to the metal-bound enzyme with a root mean square deviation of 0.4 Å between all atoms. However, the guanidinium group of Arg-33 of human PP2C*α* shifts by 1 Å due to dissociation of the phosphate ligand, suggesting that this residue is flexible and the conformation of this residue depends on the presence of the phosphate ion [2]. Although extensive structural studies suggest that this conserved arginine residue plays an important role during catalysis, it is still not well known from biochemical studies how this residue affects catalysis. Furthermore, this residue is not universally conserved but may be replaced by lysine, histidine, methionine, or alanine in some bacterial PP2C homologues (see Supplementary file in [8]). So far, only two variants (human PP2C*α* R33A [12] and tPphA R13K [13]) of this arginine residue were generated. In order to further understand the function of this conserved residue during catalysis, site-directed mutagenesis of the respective Arg-13 of PP2C phosphatase tPphA into any other amino acid residue was performed and the kinetic parameters of the variants towards different substrates were determined.

## 2. Experimental Procedures

### 2.1. Cloning, Overexpression, and Purification of tPphA Variants

The random site-directed mutagenesis of Arg-13 was carried out following the QuikChange XL (*STRATAGENE*) protocol with two complementary primers containing three random nucleotide at Arg-13 position (Table 1). The expression plasmid pET15b_tPphA (pET15b hosting the gene encoding wild type tPphA) [11] was used as the template for the construction of the *pphA* gene variants. The mutagenesis PCR was performed with *Pfu turbo* DNA polymerase using a program of 1 min at 95°C followed by 18 cycles at 95°C for 50 s, 60°C for 50 s, 68°C for 7 min, and a final extension at 68°C for 7 min. After PCR reaction, the restriction enzyme *Dpn* I was directly added to the PCR reaction tube to degrade the methylated template for 1 h at 37°C. After *Dpn* I digestion, 3 *μ*L of the PCR mixture containing the amplified pET15b_tPphA variant plasmids was transformed into *E. coli* XL-10 gold. The *E. coli* cells were grown overnight on LB (Luria-Bertani) agar plates containing 100 *μ*g/mL ampicillin. Then, 48 single colonies were picked from the LB plate and streak cultivated on new LB plates overnight. Plasmids were extracted from these 48 *E.coli* transformants by mini-prep (peqGOLD Plasmid Miniprep Kit I). The generated plasmids were transformed into *E. coli* BL21 (DE3), and the transformants were grown on LB plates containing 100 *μ*g/mL ampicillin. The next day, from each clone of transformed *E. coli* BL21 (DE3) cells, a 2 mL liquid LB medium containing 100 *μ*g/mL ampicillin was inoculated. When the O.D._600_ of the liquid cultures reached a value of 1.0–1.5, 50 *μ*L aliquots were collected and Isopropyl *β*-D-1-thiogalactopyranoside (IPTG) was added to the remaining liquid to a final concentration of 0.5 mM. After 3 hours of IPTG induction, the cells were harvested and expression of tPphA was analyzed by SDS-PAGE. All *E. coli* BL21 (DE3) transformants produced full-length tPphA variant protein. The plasmids from all 48 transformants were checked by DNA sequencing, using the T7 forward primer. DNA sequencing revealed that the 48 sequences encoded 16 different tPphA variants, whereas four possible variants (R13A, R13C, R13E, and R13V) were not obtained by this approach. The remaining variants were attempted to obtain by new rounds of site-directed mutagenesis by using the specific primers (Table 1). R13A and R13C were obtained by this means: R13A was obtained with low yield (only one clone in six trials), whereas the R13C variant was obtained immediately. Remarkably, the R13E and R13V variants could not be generated after two further site-directed mutagenesis attempts. The sequences of the generated tPphA variant plasmids are shown in Supplementary file 1 (available online at doi: 10.1155/2012/272706) and the obtained codon sequences at position 13 are summarized in Supplementary file 2. The plasmids were transformed into *E. coli* BL21 (DE3) for protein over expression and purification as described previously [11].

### 2.2. Assay of Phosphatase Activity with pNPP as Artificial Substrate

The activities of the phosphatase variants towards *p*NPP were assayed as described previously with modifications [11]. Depending on the activity of the variants, standard assays (250 *μ*L) contained 0.25 *μ*g–10 *μ*g tPphA variants in a buffer consisting of 50 mM Tris-HCl, pH 8.3, 50 mM NaCl, 1 mM DTT, and 2 mM MnCl_2_. Reactions were started by the addition of 2 mM *p*NPP at 30°C, and the increase in absorbance at 400 nm was measured in an ELx808 absorbance microplate reader (BioTek, Winooski, USA) against a blank reaction without enzyme. For *p*NPP catalytic constants, the *p*NPP concentration was varied from 0.1 mM to 10 mM. From the linear slope of each reaction, the kinetic parameters *K*_*m*_ and *V*_max⁡_ were calculated by nonlinear hyperbolic fitting according to the Michaelis-Menten equation using the GraphPad Prism 4 program (GraphPad Software Inc.).

### 2.3. Reactivity of tPphA and Its Variants towards Phosphopeptides

The activities of the phosphatase variants towards two phosphopeptides (pT-peptide, RRA(pT)VA and pS-peptide, RRA(pS)VA) were assayed as described previously [11]. In a standard assay, 0.25 *μ*g–10 *μ*g tPphA variants were reacted with 100 *μ*M phosphopeptides in a reaction volume of 100 *μ*L containing 50 mM Tris-HCl, pH 8.0, 50 mM NaCl, 2 mM MnCl_2_, and 1 mM DTT. Reactions were incubated at 30°C for 2–10 minutes, then stopped by the addition of 50 *μ*L 4.7 M HCl. The released Pi was quantified colorimetrically by the malachite-green assay [15, 16]. The absorbance of the solution at 630 nm was measured in an ELx808 absorbance microplate reader (BioTek, Winooski, USA) against a blank reaction, which was stopped at the start of the reaction by adding 50 *μ*L 4.7 M HCl. The activity of all enzymes towards peptides was calculated with a phosphate standard.

## 3. Results and Discussion

### 3.1. Site-Directed Mutagenesis of tPphA

To study the role of the Arg-13 residue in tPphA catalytic function, we attempted to replace this amino acid by any of the other 19 amino acids. For this purpose, random site-directed mutagenesis is advantageous over specific site-directed mutagenesis, since the former needs only one set of primers containing randomized base pairs at the desired codon position, whereas the latter one needs at least nineteen primers. Site-directed mutagenesis PCR was performed with a primer containing a randomized sequence at codon position 13 of the *pphA* gene and using plasmid pET15b + tPphA [11] as template. After *Dpn* I digestion and transformation (see Section 2), about 500 *E. coli* XL-10 gold colonies were grown on LB plates. 48 clones were randomly selected and plasmids were extracted from the transformants. After plasmid purification, the 48 different plasmids were transformed into *E. coli* BL21 (DE3) to induce recombinant protein synthesis. Surprisingly, all 48 transformants of *E. coli* BL21 (DE3) cells produced full-length recombinant protein of 29 kDa, meaning that no plasmid contained a stop codon at position 13. DNA sequence analysis of the mutated plasmids (Supplementary file 1) showed that sixteen different kinds of variants were acquired from this random site-directed mutagenesis approach. The codons at position 13 of these variants are shown in Supplementary file 2. The overall distribution probability of these gene codes follows the law of the gene code with the amino acids encoded by six codons having the highest possibility to be obtained (such as leucine). Four variants, R13A, R13C, R13E, R13V, were not obtained from this random site-directed mutagenesis. In order to obtain the four variants, additional site-directed mutagenesis was performed with four targeted pairs of primers (Table 1). Strikingly, five attempts to generate R13A were unsuccessful. In the sixth attempt, only one single *E. coli* XL-10 gold colony growing on the LB plate containing plasmid pET15b_R13A was obtained. Two variants, R13E and R13V, could not be generated after two attempts. The reason for the very low efficiency to generate the Arg-13 to Ala mutation and the failure to obtain Glu and Val variants could indicate a negative selection against these protein variants in *E. coli.*

### 3.2. Effect of tPphA Mutations on Catalytic Activities: pNPP Hydrolysis Activity

**Figure 1:**
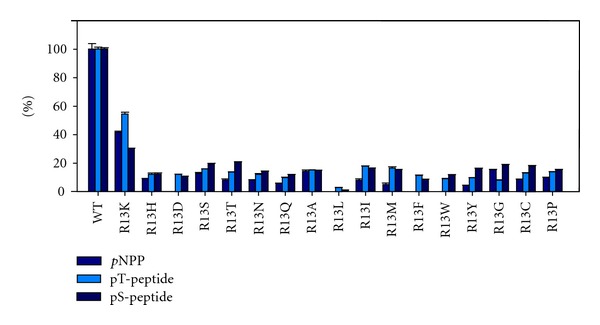
Relative activities of tPphA and Arg-13 variants, as indicated, towards different substrates. The activities of wild-type tPphA towards three substrates were set as 100% and the activities of the tPphA variants towards these substrates were adjusted accordingly. *p*NPP indicates the value for *K*_cat_/*K*_*m*_ from the *p*NPP assay (Table 2). pT-peptide indicates the relative activity with RRA(pT)VA as substrate, pS-peptide indicates the activity with RRA(pS)VA peptide as substrate (from Table 3).

All R13 variants of tPphA were produced in *E. coli* BL21 (DE3) cells and purified on His-tag affinity columns. The SDS-PAGE of the 18 purified proteins used for subsequent enzymatic assays is shown in Supplementary file 3. The tPphA variants were first tested with the artificial chromogenic substrate *p*NPP. The variant R13K displayed about 41% activity (*K*_cat_/*K*_*m*_) as compared to wild-type tPphA, which was much higher than all other Arg-13 variants (see Table 2 and Figure 1). In some bacterial PP2Cs, lysine is found at this position (see Supplementary file in [8]). The positively charged side chains of arginine and lysine can both participate in electrostatic and hydrogen bonding interactions with substrate, but with slightly different positions of the ligand. Thus, the replacement of arginine by lysine could change the specificity of enzyme. Four variants (R13L, R13F, R13W, and R13D) showed no activity towards *p*NPP. Leucine, phenylalanine, and tryptophan have long bulky hydrophobic side chains, whereas aspartate has a negatively charged carboxyl side chain. The fact that substitution of the positively charged arginine by a negatively charged residue abolishes activity confirms that this arginine residue is involved in electrostatic substrate interactions. The bulky hydrophobic side chains of leucine, phenylalanine, and tryptophan may strongly affect the structure of the catalytic core of tPphA, resulting either in complete loss of function or more specifically in inability to bind *p*NPP. In agreement with this conclusion, the tyrosine and methionine variants (R13Y and R13M), both of which are large and mainly hydrophobic, showed the lowest activity of the remaining variants, with the overall *K*_cat_/*K*_*m*_ reduced to a basal level of about 5% of wild-type activity. The other ten Arg-13 variants showed about 7–15% residual activity and 3–5 times higher *K*_*m*_ values than wild-type tPphA (Table 2). The *K*_cat_ values of R13A and R13G variants were higher than those that of the other mutant variants except R13K. Alanine and glycine have the smallest side chains, so that these substitutions may not influence the catalytic center of tPphA. Thus, *p*NPP still could be processed quite efficiently by these two variants. The *K*_cat_ value of the R13P variant is only twofold lower that wild-type tPphA. Proline has a cyclic structure and a fixed **φ** angle. Arg-13 is located at a *β*-turn connecting *β*1 and *β*2 sheets. Probably, proline at this position does not affect this *β*-turn. Together, these results show that R13 is not absolutely essential for catalysis, but that it is optimal for the reaction by positively affecting *K*_*m*_ and *K*_cat_  for substrate binding and turnover. Negative charge is not permitted and hydrophobic bulky side-chains are inhibitory. As long as the space occupied by Arg-13 remains free (Gly and Ala variants), an appreciable catalysis can take place.

### 3.3. Reactivity of tPphA and Its Variants towards Phosphopeptides

To find out whether the observed defects in *p*NPP dephosphorylation are specific for this substrate or reflect general impairment of catalysis, the variants were also tested with two different phosphopeptides (RRA(pT)VA and RRA(pS)VA) as substrate. Strikingly, all variants could dephosphorylate the two phosphopeptides, even when they were inactive towards *p*NPP (R13W, R13L, R13F, R13D) (Table 3, Figure 1). This shows that the phosphopeptides recovered to a small but measurable extent the catalytic activity in variants R13W, R13L, R13F, and R13D. The R13L variant had the lowest activity towards phosphopeptides. The long aliphatic side chain of leucine may interfere directly with the catalytic centre of tPphA, impairing enzyme activity regardless of the substrate. The fact that R13D recovered some activity towards phosphopeptides was unexpected. The carboxyl group of aspartate was expected to exclude the substrate phosphate from the catalytic site. The gain of activity using phosphopeptides compared to *p*NPP as substrate might be achieved by a substrate-induced movement of the aspartate side-chain. All together, the recovery of some residual activity by using phosphopeptides as compared to *p*NPP in various variants indicates that the five residues neigbouring the phosphorylated residues (pT and pS) help anchoring the phosphothreonyl- or phosphoseryl-residues to the catalytic center. After anchoring, the phosphopeptides may affect the conformation of the catalytic centers of tPphA variants in a way that productive enzyme-substrate complexes are formed and the variants can dephosphorylate these two peptides. This assumption agrees with previous findings, where we showed that the free phosphorylated amino acid phospho-serine and the tripeptide (G(pS)E) cannot be dephosphorylated by tPphA, whereas the hexapeptides can be dephosphorylated [11]. This indicates that neighbouring interactions of the phosphorylated residue are required for catalysis, either for proper binding or turnover of the substrate.

### 3.4. The Function of Arg-13 Residue of tPphA

In the crystal structures of various PP2C homologues, the long guanidinium side chain of the conserved arginine was hydrogen bonded with phosphate ions or analogs in different conformations, suggesting that the arginyl-side chain anchors the substrate phosphate at various stages of catalysis. Whether this interaction is necessary for catalysis was not clearly resolved. In this study, the kinetic parameters of tPphA Arg-13 variants towards *p*NPP are consistent with the suggestion of substrate binding, since all the variants showed higher *K*_*m*_ values than wild-type tPphA. Furthermore, the decrease in *K*_cat_ value indicates a role in substrate turn-over. Enzymatic characterization of human PP2C*α* reactivity towards *p*NPP indicated that the phosphate release from this enzyme is the rate-limiting step of catalysis [17]. The different conformations of the arginyl-side chain observed in PP2C crystal structures indicate that this arginine residue could guide the phosphate group through the catalytic cycle. The guanidinium side chain of this arginine residue together with the metal center could further play a role in stabilizing the transition state of the substrate phosphate. In the absence of the conserved arginine residue, the metal ions in the catalytic center of PP2C can still stabilize the transition state of the phosphate and break the covalent bond between phosphate and the leaving group, but with decreased catalytic efficiency. Following hydrolysis, the arginyl-side chain could drag the phosphate ion from the catalytic center by its guanidium side chain followed by release of the phosphate ion. This suggestion agrees with the observed properties of the R13 variants: replacement of the arginyl-side chain by lysine has only a modest effect on both *K*_cat_ and *K*_*m*_, indicating that the shorter lysine side chain can partially replace the function of the arginyl-side chain. However, when the arginyl-residue is replaced by any neutral residue, the reaction is impaired more strongly, with both *K*_*m*_ and *K*_cat_ being affected. In these variants, the substrate phosphate is not assisted by the arginyl-side chain during catalysis; therefore, it binds weaker and is released more slowly from the catalytic centre. The difference between *p*NPP hydrolysis and phospho-peptide dephosphorylation reflects the role of the neighbouring amino acids for substrate binding. The failure of the R13W, R13F, and R13L variants to dephosphorylate *p*NPP is, therefore, more likely due to poor binding of *p*NPP than to impaired turn-over. Those rare PP2C homologues, in which this conserved arginine residue is substituted by other residues, are expected to display diminished catalytic activity. The activity of the PP2C homologues can thus be adapted in different organisms and signal transduction pathways towards fast or slow signal response by evolutionary changes in the position of the conserved Arg residue.

The molecular mechanism of PP2Cs becomes more and more clear, but there are still obvious open questions about this type of protein phosphatase, such as the precise mechanism of protein substrate recognition and how PP2Cs are regulated. More biochemical studies of and new methods are necessary to solve these issues.

## Supplementary Material

Summary of the random site-directed mutagenesis of tPphA at codon position 13. The codons of the at position 13 of variant amino acids replacing wild type tPphA Arg-13 are given. The full sequences of the variants are given in Supplementary file 1.
DNA sequence analysis of the mutated plasmids encoding 16 different tPphA variants, as summarized in Supplementary file 2.
Electrophoretic analysis of the 18 purified tPphA variants used in this study as shown by SDS-PAGE and Coomassie staining.

## Figures and Tables

**Table 1:** Primers used for PCR amplification of tPphA and for site-directed mutagenesis.

T7	5′; 5′-TAATACGACTCACTATAGGG-3′
	3′; 5′-GCTAGTTATTGCTCAGCGG-3′
R13X	5′; 5′-CTGACTGTGGTCTGATTNNNAAAAGCAATCAGGATGC-3′
	3′; 5′-GCATCCTGATTGCTTTTNNNAATCAGACCACAGTCAG-3′
R13A	5′; 5′-CTGACTGTGGTCTGATTGCTAAAAGCAATCAGGATGC-3′
	3′; 5′-GCATCCTGATTGCTTTTAGCAATCAGACCACAGTCAG-3′
R13C	5′; 5′-CTGACTGTGGTCTGATTTGTAAAAGCAATCAGGATGC-3′
	3′; 5′-GCATCCTGATTGCTTTTACAAATCAGACCACAGTCAG-3′
R13E	5′; 5′-CTGACTGTGGTCTGATTGAAAAAAGCAATCAGGATGC-3′
	3′; 5′-GCATCCTGATTGCTTTTTTCAATCAGACCACAGTCAG-3′
R13V	5′; 5′-CTGACTGTGGTCTGATTGTTAAAAGCAATCAGGATGC-3′
	3′; 5′-GCATCCTGATTGCTTTTAACAATCAGACCACAGTCAG-3′

**Table 2:** Kinetic parameters of tPphA and Arg-13 variants towards *p*NPP. *p*NPP assays were carried out as described in Experimental Procedures (Subsection 2.2). The pH of the reaction was 8.3. From the apparent reaction velocities of three independent repetitions, the kinetic parameters were calculated by linear fitting using the program GraphPad Prism 4. ± indicates standard error. The results were obtained from single preparations of the wild-type (WT) and variant tPphA purifications.

Classification of Arg-13 variants	Variants	*K*_*m*_ [mM] (*p*NPP)	*K*_cat_ [s^−1^] *p*NPP	*K*_cat_/*K*_*m*_ [s^-1 ^M^−1^]
Arg-13 changed to residues with electrically charged side chains	WT(R13R)	1.06 ± 0.05	2.25 ± 0.10	2122 ± 94
	R13K	1.39 ± 0.10	1.22 ± 0.03	878 ± 21
	R13H	3.30 ± 0.17	0.64 ± 0.01	194 ± 3
	R13D	—	—	—

Arg-13 changed to residues with polar uncharged side chains	R13S	3.85 ± 0.32	1.06 ± 0.04	275 ± 10
	R13T	5.37 ± 1.00	0.94 ± 0.08	175 ± 15
	R13N	4.73 ± 0.53	0.81 ± 0.04	171 ± 8
	R13Q	4.06 ± 0.21	0.50 ± 0.01	123 ± 2

Arg-13 changed to residues with hydrophobic side chains	R13A	5.11 ± 0.23	1.56 ± 0.03	305 ± 6
	R13L	—	—	—
	R13I	4.58 ± 0.54	0.75 ± 0.04	164 ± 9
	R13M	7.36 ± 2.76	0.76 ± 0.15	103 ± 20

Arg-13 changed to residues with aromatic side chains	R13F	—	—	—
	R13W	—	—	—
	R13Y	4.31 ± 0.28	0.42 ± 0.01	97 ± 2

Arg-13 changed to residues with special side chains	R13G	3.58 ± 0.22	1.17 ± 0.03	327 ± 8
	R13C	4.27 ± 0.39	0.79 ± 0.01	185 ± 2
	R13P	4.77 ± 0.17	1.01 ± 0.02	212 ± 4

—: not detectable.

**Table 3:** The specific activity of tPphA and Arg-13 variants towards three phosphopeptides. Reactions were performed in the buffer as described in Experimental Procedures (Subsection 2.3). Triplicate assays were used. ± indicates the standard error. The results were obtained from single preparations of the wild-type (WT) and variant tPphA purifications.

Classification of Arg-13 variants	Variants	Thr peptide nmol/min/*μ*g	Ser peptide nmol/min/*μ*g
Arg-13 changed to residues with electrically charged side chains	WT(R13R)	9.32 ± 0.14	5.49 ± 0.06
	R13K	5.09 ± 0.10	1.67 ± 0.01
	R13H	1.13 ± 0.08	0.68 ± 0.04
	R13D	1.12 ± 0.03	0.59 ± 0.01

Arg-13 changed to residues with polar uncharged side chains	R13S	1.47 ± 0.02	1.07 ± 0.02
	R13T	1.28 ± 0.02	1.13 ± 0.02
	R13N	1.13 ± 0.06	0.78 ± 0.03
	R13Q	0.91 ± 0.05	0.65 ± 0.03

Arg-13 changed to residues with hydrophobic side chains	R13A	1.40 ± 0.02	0.81 ± 0.01
	R13L	0.26 ± 0.02	0.05 ± 0.01
	R13I	1.67 ± 0.01	0.90 ± 0.01
	R13M	1.55 ± 0.07	0.84 ± 0.01

Arg-13 changed to residues with aromatic side chains	R13F	1.07 ± 0.04	0.47 ± 0.01
	R13W	0.84 ± 0.03	0.65 ± 0.01
	R13Y	0.91 ± 0.01	0.89 ± 0.02

Arg-13 changed to residues with special side chains	R13G	0.74 ± 0.04	1.04 ± 0.02
	R13C	1.21 ± 0.03	0.98 ± 0.03
	R13P	1.29 ± 0.03	0.85 ± 0.02
