# Supplementary material for: The Role of Arg13 in Protein Phosphatase M tPphA from Thermosynechococcus elongatus
Source: Enzyme Res. 2012 Jun 6;2012:272706. doi: 10.1155/2012/272706 (PMC3375153; doi:10.1155/2012/272706)
Supplement: Supplementary file 1 — Summary of the random site-directed mutagenesis of tPphA at codon position 13. The codons of the at position 13 of variant amino acids replacing wild type tPphA Arg-13 are given. The full sequences of the variants are given in Supplementary file 1. DNA sequence analysis of the mutated plasmids encoding 16 different tPphA variants, as summarized in Supplementary file 2. Electrophoretic analysis of the 18 purified tPphA variants used in this study as shown by SDS-PAGE and Coomassie staining. [file 272706.f1.pdf]

Supplementary file 1: DNA DNA sequencing files

R13L

A6

>7951850.seq - ID: A6-T7 on 2011/5/21-8:50:45 automatically edited with PhredPhrap, start with base no.: 7 Internal Params: Windowsize: 20, Goodqual: 19, Badqual: 10, Minseqlength: 50, nbadelimit: 1

gaTaATTCCcTCtnanaaATTTtGTTtAaCTTtAAGAAGGAGaTATACCATgGgCAG
CAGCCnncctcatcatcATCACAGCAGCGGCCTggtGCCGCGCGGCAGCCATATGG
ACGTTGCTGGCTTAACTGACTGTGGTCTGATTCTTAAAAGCAATCAGGATG
CTTTTTATATTGATGAAAAGCATCAGCGCTTTTTATTGTTGCAGATGGCA
TGGGGGGACACGCCGGCGGCGaGGAAGCCAGTCGTTTAGCCGTCGATCAC
ATTCGGCAGTATTTGGAAACCCACCTTGAAGACCTGCAGCACGACCCCGT
CACTCTACTCCGCCAAGCCTTTCTTGCAGCCAATCATGCTATTGTTGAGCA
GCAGCGCCAAAACAGTGCCCGTGCCGATATGGGTACGACGGCTGTGGTGA
TTCTACTTGATGAAAAGGGCGATCGCGCTTGGTGTGCCCATGTGGGCGAC
TCCCACATCTACCGCTGGCGCAAAGATCAACTCCAGCAGATTACCAGCGA
CCACACGTGGATTGCTCAAGCCGTGCAACTGGGTAGCTTGACCATTGAGC
AGGCGCGGCAGCATCCGTGGCGCCATGTGCTCTCCCAGTGTTTAGGACGC
GAGGACCTCAGCCAAATTGATATTCAGCCCATCGACCTAGAGCCGGGCGA
TCGCCTGCTGCTGTGCAGTGATGGCCTGACCGAAGAACTCACAGATGACG
TCATCAGTATCTACCTCAGTGAACCCAATGTTCAAAAAGCTGCTGCTGCCC
TTGTGGATGCAGCCAAAACCCATGGCGgGCGTGACAATGTCACCGTCGTT
GTCATCAGTgTTTAacCGCTCgaGGATCCggcTGCTAACAAAGCCCCGAaagGA
AGCTgaantTGGCTGCtnncnccngCTgaaGcAATAACtaaGCATaaCCCCCTTggggnc
CTCTAAAACGGGGTcttTGaagGgGTTtTTttGCttganagggnngGAACTAnnntccCgga
nna

C3

>7951861.seq - ID: C3-T7 on 2011/5/21-8:50:45 automatically edited with PhredPhrap, start with base no.: 20 Internal Params: Windowsize: 20, Goodqual: 19, Badqual: 10, Minseqlength: 50, nbadelimit: 1

AaanaTTTtGTttaCTTtAaGAAGGAGATATACCATGGGCAGCAGCCATCATCAT
CATCATCACAGCAGCGGCCTggtGCCGCGCGGCAGCCATATGGACGTTGCtG
GCTTAAcTGACTgtggtcTGATTCTCAAAAGCAATCAGGATGCTTTTTATATTG
ATGAAAAGCATCAGCGCTTTTTATTGTTGCAGATGGCATGGGGGGACAC
GCCGGCGGcgagGAAGCCAGTCGTTTAGCCgtcGATCACATTCGGCAGTATTT
GGAAACCCACCTtGAAGACCTGCAGCACGACCCCGTCACTCTACTCCGCCA
AGCCTTTCtTGCAGCCAATCATGCTATTGTTGAGCAGCAGCGCCAAAACAG
TGCCCGTGCCGATATGGGTACGACGGCTGTGGTGATTCTACTTGATGAAA
AGGGCGATCGCGCtTGGTGTGCCCATGTGGGCGACTCCCGCATCTACCGCT
GGCGCAAAGATCAACTCCAGCAGATTACCAGCGACCACACGTGGATTGCT
CAAGCCGTGCAACTGGGTAGCTTGACCATTGAGCAGGCGCGGCAGCATCC

GTGGCGCCATGTGCTCTCCCAGTGTTTAGGACGCGAGGACCTCAGCCAAA
TTGATATTCAGCCCATCGACCTAGAGCCGGGCGATCGCCTGCTGCTGTGC
AGTGATGGCCTGACCGAAGAACTCACAGATGACGTCATCAGTATCTACCT
CAGTGAACCCAATGTTCAAAAAGCTGCTGCTGCCCTTGTGGATGCAGCCA
AAACCCATGGCGGGCGTGACAATGTCACCGTCGTTGTCATCAGTGTTTAA
CCGCTCGAGGATCCGGCTGCTAACAAAGCCCgaAAGGAAGCTGAGTTGGC
TGCTGCCACCGCTGAGCAATAACTAGCATAACCcCTTGGGGCCTCTAAACG
GGTCTTGAGGgGTtTTTTGCTGAAAGGAGGAACTATATCCGGATATCCCGC
AAGaggCCCGGCAGTACcGGCATAAccAaGCCTATGCCTAcAGCATCCaaGGG
TGAcgGtnc

E6

>8055299.seq - ID: E6-T7 on 2011/6/15-10:18:43 automatically edited with PhredPhrap, start with base no.: 10 Internal Params: Windowsize: 20, Goodqual: 19, Badqual: 10, Minseqlength: 50, nbadelimit: 1

atTCCCTcTnnAataTTTTGTTTACTTtAaGAAGGAGATATACCATGGGCAGCAG
CCATCATCATCATCATCACAGCAGCGGCCTGGTGCCGCGCGGCAGCCATA
TGGACGTTGCTGGCTTAACTGACTGTGGTCTGATTTTAAAAAGCAATCAGG
ATGCTTTTTATATTGATGAAAAGCATCAGCGCTTTTTTATTGTTGCAGATG
GCATGGGGGGACACGCCGGCGGCGAGGAAGCCAGTCGTTTAGCCGTCGA
TCACATTCGGCAGTATTTGGAAACCCACCTTGAAGACCTGCAGCACGACC
CCGTCACTCTACTCCGCCAAGCCTTTCTTGCAGCCAATCATGCTATTGTTG
AGCAGCAGCGCCAAAACAGTGCCCGTGCCGATATGGGTACGACGGCTGTG
GTGATTCTACTTGATGAAAAGGGCGATCGCGCTTGGTGTGCCCATGTGGG
CGACTCCCGCATCTACCGCTGGCGCAAAGATCAACTCCAGCAGATTACCA
GCGACCACACGTGGATTGCTCAAGCCGTGCAACTGGGTAGCTTGACCATT
GAGCAGGCGCGGCAGCATCCGTGGCGCCATGTGCTCTCCCAGTGTTTAGG
ACGCGAGGACCTCAGCCAAATTGATATTCAGCCCATCGACCTAGAGCCGG
GCGATCGCCTGCTGCTGTGCAGTGATGGCCTGACCGAAGAACTCACAGAT
GACGTCATCAGTATCTACCTCAGTGAACCCAATGTTCAAAAAGCTGCTGC
TGCCCTTGTGGATGCAGCCAAAACCCATGGCGGGCGTGACAATGTCACCG
TCGTTGTCATCAGTGTTTAACCGCTCGAGGATCCGGCTGCTAACAAAGCCC
GaAaGGAAGCTGAGTTGGCTGCTGCCACCGCTGancAATAACTagCATAACcc
cTTGGGGCCTCTAAACGGGTcTTGAGGgGTTTTTTGCTGAAAGGagGAACTat
aTCCGGataTCCCGCannAGGCCCGGCAGTACCGGCATAACCAAGcCTATGCC
TACAGCATCCAGGGtGACggtGCCgannatgacgannaanncATtGTTa

E7

>8055300.seq - ID: E7-T7 on 2011/6/15-10:18:47 automatically edited with PhredPhrap, start with base no.: 15 Internal Params: Windowsize: 20, Goodqual: 19, Badqual: 10, Minseqlength: 50, nbadelimit: 1

TCCCTctnaATaTTTTGTTtACTTtAAGaAGGAnaaTACCATGGgCAGCAGCCATC
ATCATCATCATCACAGCAGCGGCCTGgtGCCGCGCGGCAGCCATATGGACG
TTGCTGGCTTAACTGACTGTGGTCTGATTCTTAAAAGCAATCAGGATGCTT
TTTATATTGATGAAAAGCATCAGCGCTTTTTTATTGTTGCAGATGGCATGG
GGGGACACGCCGGCGGCGAGGAAGCCAGTCGTTTAGCCGTCGATCACATT

CGGCAGTATTTGGAAACCCACCTTGAAGACCTGCAGCACGACCCCGTCAC
TCTACTCCGCCAAGCCTTTCTTGCAGCCAATCATGCTATTGTTGAGCAGCA
GCGCCAAAACAGTGCCCGTGCCGATATGGGTACGACGGCTGTGGTGATTC
TACTTGATGAAAAGGGCGATCGCGCTTGGTGTGCCCATGTGGGCGACTCC
CGCATCTACCGCTGGCGCAAAGATCAACTCCAGCAGATTACCAGCGACCA
CACGTGGATTGCTCAAGCCGTGCAACTGGGTAGCTTGAccaTTGAGCAGGC
GCGGcagCATCCGTGGCGCCATGTGCTCtnccAGTGTTTAGgACGCGAGGACC
TCaGccaAatTGAtattcAGCCCATCgaccTAgagCCGGGCGatcgcCTGCTGCTgngcA
GTGATGGCCTnancGAAGAactCacaGATGAcntcATCantntctacncTcatGAAcccATG
TTCAAAannTGCTGCTgcctTGTGgatncacCaaanCCATGGCggnct

F1

>8055302.seq - ID: F1-T7 on 2011/6/15-10:18:43 automatically edited with PhredPhrap, start with base no.: 10 Internal Params: Windowsize: 20, Goodqual: 19, Badqual: 10, Minseqlength: 50, nbadelimit: 1

atTCCCTcTnnaataTTTTGTTTACTTtAaGAAGGAGATATACCATGGGCAGCAG
CCATCATCATCATCATCACAGCAGCGGCCTGGTGCCGCGCGGCAGCCATA
TGGACGTTGCTGGCTTAACTGACTGTGGTCTGATTCTCAAAAGCAATCAG
GATGCTTTTTATATTGATGAAAAGCATCAGCGCTTTTTTATTGTTGCAGAT
GGCATGGGGGGACACGCCGGCGGCGAGGAAGCCAGTCGTTTAGCCGTCG
ATCACATTCGGCAGTATTTGGAAACCCACCTTGAAGACCTGCAGCACGAC
CCCGTCACTCTACTCCGCCAAGCCTTTCTTGCAGCCAATCATGCTATTGTT
GAGCAGCAGCGCCAAAACAGTGCCCGTGCCGATATGGGTACGACGGCTGT
GGTGATTCTACTTGATGAAAAGGGCGATCGCGCTTGGTGTGCCCATGTGG
GCGACTCCCGCATCTACCGCTGGCGCAAAGATCAACTCCAGCAGATTACC
AGCGACCACACGTGGATTGCTCAAGCCGTGCAACTGGGTAGCTTGACCAT
TGAGCAGGCGCGGCAGCATCCGTGGCGCCATGTGCTCTCCCAGTGTTTAG
GACGCGAGGACCTCAGCCAAATTGATATTCAGCCCATCGACCTAGAGCCG
GGCGATCGCCTGCTGCTGTGCAGTGATGGCCTGACCGAAGAACTCACAGA
TGACGTCATCAGTATCTACCTCAGTGAACCCAATGTTCAnAAAGCTGCTGC
TGCCCTTGTGGATGCAGCCAAAACCCATGGCGGGCGTGACAATGTCACCG
TCGTTGTCATCAGTGTTTAACCGCTCGAGGATCCGGCTGCTAACAnAGCCC
GAAAGGAAGCTGagtTGGCTGCTGCCACCGCTGancAATAACTAGCATAACC
CCTTGGGGCCtCTAAACGGGTCTTGAGGGGtTTTTTGCTGAAAGGAGGAAC
TATATccgGAtATCCCGCaanagGCccggCAGTACCgGCataACCAAGCCTATGCC
TanagCATCCaaggg

F5

>8055306.seq - ID: F5-T7 on 2011/6/15-10:18:43 automatically edited with PhredPhrap, start with base no.: 14 Internal Params: Windowsize: 20, Goodqual: 19, Badqual: 10, Minseqlength: 50, nbadelimit: 1

TTCCcTcnnaaaTaATTTTGTTtACTTtAAGAAGGAGATATACCATGGGCAGCA
GCCATCATCATCATCATCACAGCAGCGGCCTGGTGCCGCGCGGCAGCCAT
ATGGACGTTGCTGGCTTAACTGACTGTGGTCTGATTTTAAAAAGCAATCAG
GATGCTTTTTATATTGATGAAAAGCATCAGCGCTTTTTTATTGTTGCAGAT
GGCATGGGGGGACACGCCGGCGGCGAGGAAGCCAGTCGTTTAGCCGTCG

ATCACATTCGGCAGTATTTGGAAACCCACCTTGAAGACCTGCAGCACGAC
CCCGTCACTCTACTCCGCCAAGCCTTTCTTGCAGCCAATCATGCTATTGTT
GAGCAGCAGCGCCAAAACAGTGCCCGTGCCGATATGGGTACGACGGCTGT
GGTGATTCTACTTGATGAAAAGGGCGATCGCGCTTGGTGTGCCCATGTGG
GCGACTCCCGCATCTACCGCTGGCGCAAAGATCAACTCCAGCAGATTACC
AGCGACCACACGTGGATTGCTCAAGCCGTGCAACTGGGTAGCTTGACCAT
TGAGCAGGCGCGGCAGCATCCGTGGCGCCATGTGCTCTCCCAGTGTTTAG
GACGCGAGGACCTCAGCCAAATTGATATTCAGCCCATCGACCTAGAGCCG
GGCGATCGCCTGCTGCTGTGCAGTGATGGCCTGACCGAAGAACTCACAGA
TGACGTCATCAGTATCTACCTCAGTGAACCCAATGTTCAAnAAGCTGCTGC
TGCCCTTGTGGATGCAGCCAAAACCCATGGCGGGCGTGACAATGTCACCG
TCGTTGTCATCAGTGTTTAACCGCTCGAGGATCCGGCTGCTAACAAAGCcC
GAAAGGAAGCtgaGTTGGCTGCTGCCACcGcTGancnataacTAGCAtAACCCCCT
TGGgggcCTCTaaacGGGgtc

F8

>8055309.seq - ID: F8-T7 on 2011/6/15-10:18:47 automatically edited with PhredPhrap, start with base no.: 22 Internal Params: Windowsize: 20, Goodqual: 19, Badqual: 10, Minseqlength: 50, nbadelimit: 1

annaTTTTGTttaCTTtAaGAAGGAGATATACCATGGGCAGCAGCCATCATCAT
CATCATCACAGCAGCGGCCTGGTGCCGCGCGGCAGCCATATGGACGTTGC
TGGCTTAACTGACTGTGGTCTGATTCTTAAAAGCAATCAGGATGCTTTTTA
TATTGATGAAAAGCATCAGCGCTTTTTTATTGTTGCAGATGGCATGGGGGG
ACACGCCGGCGGCGAGGAAGCCAGTCGTTTAGCCGTCGATCACATTCGGC
AGTATTTGGAAACCCACCTTGAAGACCTGCAGCACGACCCCGTCACTCTA
CTCCGCCAAGCCTTTCTTGCAGCCAATCATGCTATTGTTGAGCAGCAGCGC
CAAAACAGTGCCCGTGCCGATATGGGTACGACGGCTGTGGTGATTCTACT
TGATGAAAAGGGCGATCGCGCTTGGTGTGCCCATGTGGGCGACTCCCGCA
TCTACCGCTGGCGCAAAGATCAACTCCAGCAGATTACCAGCGACCACACG
TGGATTGCTCAAGCCGTGCAACTGGGTAGCTTGACCATTGAGCAGGCGCG
GCAGCATCCGTGGCGCCATGTGCTCTCCCAGTGTTTAGGACGCGAGGACC
TCAGCCAAATTGATATTCAGCCCATCGACCTAGAGCCGGGCGATCGCCTG
CTGCTGTGCAGTGATGGCCTGACCGAAGAACTCACAGATGACGTCATCAG
TATCTACCTCAGTGAACCCAATGTTCAAAnAGCTGCTGCTGCCCTTGTGGA
TGCAGCCAAnACCCATGGCGGGCGTGACAATGTCACCGTCGTTGTCATCA
GTGTTTAACCGCTCGAGGATCCGGCTGCTAACAAAGCCCGanaGGAAGCTG
AGTTGGCTGCTgccACCGCTGAGCAATAACTAGCATAaCCCCTTGgGGCCTC
TAAACGGGTCTTGAGGgGttTTTTGcTGaAaggaggAACTannnncCGGAtATCCCG
caaannGGCCCGGGCAGTACcgggcannaaccaa

R13R

B1

>7951853.seq - ID: B1-T7 on 2011/5/21-8:12:39 automatically edited with PhredPhrap, start with base no.: 20 Internal Params: Windowsize: 20, Goodqual: 19, Badqual: 10, Minseqlength: 50, nbadelimit: 1

annaTTTtGTTtACTTtAaGAAGGAGATATACCATGGGCAGCAGCCATCATCAT

CATCATCACAGCAGCGGCCTGGTGCCGCGCGGCAGCCATATGGACGTTGC
TGGCTTAACTGACTgtGGTCTGATTCGCAAAAGCAATCAGGATGCTTTTTAT
ATTGATGAAAAGCATCAGCGCTTTTTATTGTTGCAGATGGCATGGGGGG
ACACGCCGGCGGCGAGGAAGCCAGTCGTTTAGCCGTCGATCACATTCGGC
AGTATTTGGAAACCCACCTTGAAGACCTGCAGCACGACCCCGTCACTCTA
CTCCGCCAAGCCTTTCTTGCAGCCAATCATGCTATTGTTGAGCAGCAGCGC
CAAAACAGTGCCCGTGCCGATATGGGTACGACGGCTGTGGTGATTCTACT
TGATGAAAAGGGCGATCGCGCTTGGTGTGCCCATGTGGGCGACTCCCGCA
TCTACCGCTGGCGCAAAGATCAACTCCAGCAGATTACCAGCGACCACACG
TGGATTGCTCAAGCCGTGCAACTGGGTAGCTTGACCATTGAGCAGGCGCG
GCAGCATCCGTGGCGCCATGTGCTCTCCCAGTGTTTAGGACGCGAGGACC
TCAGCCAAATTGATATTCAGCCCATCGACCTAGAGCCGGGCGATCGCCTG
CTGCTGTGCAGTGATGGCCTGACCGAAGAACTCACAGATGACGTCATCAG
TATCTACCTCAGTGAACCCAATGTTCAAAAAGCTGCTGCTGCCCTTGTGGA
TGCAGCCAAAACCCATGGCGGGCGTGACAATGTCACCGTCGTTGTCATCA
GTGTTTAACCGCTCGAGGATCCGGCTGCTAACAAAGCCCGAAAGGAAGCT
GAGTTGGCTGCTGCCACCGCTGAGCAATAACTAGCATAACCCCTTGGGGC
CTCTAAACGGGTCTTGAGGGGTTTTTTGCTGAAAGGAGGAACTATATCCG
GATATCCCGCAAGAGGCCCGGCAGTACCGGCATAACCAAGCCTATGCCTA
CAGCATCCAGGGTGACGGTGCCGAGGATGAcgATGAGcGCATTGTTAGATT
tCATACACGGTGCCTGACTGCGTTAGCAATTTAACTgtgatnAACTACcGCATT
AAaGCTTATcGaTganna

C8

>7951866.seq - ID: C8-T7 on 2011/5/21-8:12:44 automatically edited with PhredPhrap, start with base no.: 17 Internal Params: Windowsize: 20, Goodqual: 19, Badqual: 10, Minseqlength: 50, nbadelimit: 1

TnnaataTTTtGTTTACTTtAaGAAGGAGATATACCATGGGCAGCAGCCATcnnc
ATCATCATCACAGCAGCGGCCTGgtGCCGCGCGGCAGCCATATGGACGTTG
CTGGCTTAACTGACTGTGGTCTGATTCGTAAAAGCAATCAGGATGCTTTTT
ATATTGATGAAAAGCATCAGCGCTTTTTATTGTTGCAGATGGCATGGGGG
GACACGCCGGCGGCGAGGAAGCCAGTCGTTTAGCCGTCGATCACATTCGG
CAGTATTTGGAAACCCACCTTGAAGACCTGCAGCACGACCCCGTCACTCT
ACTCCGCCAAGCCTTTCTTGCAGCCAATCATGCTATTGTTGAGCAGCAGCG
CCAAAACAGTGCCCGTGCCGATATGGGTACGACGGCTGTGGTGATTCTAC
TTGATGAAAAGGGCGATCGCGCTTGGTGTGCCCATGTGGGCGACTCCCGC
ATCTACCGCTGGCGCAAAGATCAACTCCAGCAGATTACCAGCGACCACAC
GTGGATTGCTCAAGCCGTGCAACTGGGTAGCTTGACCATTGAGCAGGCGC
GGCAGCATCCGTGGCGCCATGTGCTCTCCCAGTGTTTAGGACGCGAGGAC
CTCAGCCAAATTGATATTCAGCCCATCGACCTAGAGCCGGGCGATCGCCT
GCTGCTGTGCAGTGATGGCCTGACCGAAGAACTCACAGATGACGTCATCA
GTATCTACCTCAGTGAACCCAATGTTCAAAAAGCTGCTGCTGCCCTTGTGG
ATGCAGCCAAnACCCATGGCGGGCGTGACAATGTCACCGTCGTTGTCATC
AGTGTTTAACCGCTCGAGGATCCGGCTGCTAACAAAGCCCGAAAGGAAGC
TGAGTTGGCTGCTGCCACCGCTGAGCAATAACTAGCATAACCCCTTGGGG

CCTCTAAACGGGTCTTGAGGgGTTTTTGCTGAAAGGAGGAACTATATCCG
GATATCCCGCAAgAGGCCCGGCAGTACCGGCATAACCAAGCCTATGCCTA
CAGCATCCAGGGTGACGGTGCCGAGGATGACGATGancGCATTGTTAgaTTT
CATACACGGtgCCTGACTGCGTTAGCAATTTAACTnngataAACTACCgCATTA
AagCTTATcGaTgnnaa

E3

>8055296.seq - ID: E3-T7 on 2011/6/15-10:18:43 automatically edited with PhredPhrap, start with base no.: 11 Internal Params: Windowsize: 20, Goodqual: 19, Badqual: 10, Minseqlength: 50, nbadelimit: 1

aaTTCCcTCtnaaAnaATTTTGTTtaACTTtAAGAAGGAGATATACCATGGgCAGC
AGCCATCATCATCATCATCACAGCAGCGGCCTGGTGCCGCGCGGCAGCCA
TATGGACGTTGCTGGCTTAACTGACTGTGGTCTGATTCGCAAAAGCAATC
AGGATGCTTTTTATATTGATGAAAAGCATCAGCGCTTTTTTATTGTTGCAG
ATGGCATGGGGGGACACGCCGGCGGCGAGGAAGCCAGTCGTTTAGCCGT
CGATCACATTCGGCAGTATTTGGAAACCCACCTTGAAGACCTGCAGCACG
ACCCCGTCACTCTACTCCGCCAAGCCTTTCTTGCAGCCAATCATGCTATTG
TTGAGCAGCAGCGCCAAAACAGTGCCCGTGCCGATATGGGTACGACGGCT
GTGGTGATTCTACTTGATGAAAAGGGCGATCGCGCTTGGTGTGCCCATGT
GGGCGACTCCCGCATCTACCGCTGGCGCAAAGATCAACTCCAGCAGATTA
CCAGCGACCACACGTGGATTGCTCAAGCCGTGCAACTGGGTAGCTTGACC
ATTGAGCAGGCGCGGCAGCATCCGTGGCGCCATGTgCTCTCCCAGtGtttAGG
ACgCgaGGAcctCaGcCAAattGaTaTTCagcCcaTcgaCCTagaGCCGGgcgATCGcctgc
TgcTgtgCagtGATgGCCTGAccgAAGAACtCACagatgaCgtCATCagt

F3

>8055304.seq - ID: F3-T7 on 2011/6/15-8:25:26 automatically edited with PhredPhrap, start with base no.: 27 Internal Params: Windowsize: 20, Goodqual: 19, Badqual: 10, Minseqlength: 50, nbadelimit: 1

AATTTTgTTTAACTTTAAGAAGGAGAtATACCATGGGCAGCAGCCATCATC
ATCATCATCACAGCAGCGGCCTGGTGCCGCGCGGCAGCCATATGGACGTT
GCTGGCTTAACTGACTGTGGTCTGATTCGTAAAAGCAATCAGGATGCTTTT
TATATTGATGAAAAGCATCAGCGCTTTTTTATTGTTGCAGATGGCATGGGG
GGACACGCCGGCGGCGAGGAAGCCAGTCGTTTAGCCGTCGATCACATTCG
GCAGTATTTGGAAACCCACCTTGAAGACCTGCAGCACGACCCCGTCACTC
TACTCCGCCAAGCCTTTCTTGCAGCCAATCATGCTATTGTTGAGCAGCAGC
GCCAAAACAGTGCCCGTGCCGATATGGGTACGACGGCTGTGGTGATTCTA
CTTGATGAAAAGGGCGATCGCGCTTGGTGTGCCCATGTGGGCGACTCCCG
CATCTACCGCTGGCGCAAAGATCAACTCCAGCAGATTACCAGCGACCACA
CGTGGATTGCTCAAGCCGTGCAACTGGGTAGCTTGACCATTGAGCAGGCG
CGGCAGCATCCGTGGCGCCATGTGCTCTCCCAGTGTTTAGGACGCGAGGA
CCTCAGCCAAATTGATATTCAGCCCATCGACCTAGAGCCGGGCGATCGCC
TGCTGCTGTGCAGTGATGGCCTGACCGAAGAACTCACAGATGAcGTCATC
AGTATCTACCTCAGTGAACCCAATGTTCAAAAAGCTGCTGCTGCCCTTGTG
GATGCAGCCaAAACCCATGGCGGGCGTGACAATGTCACCGTCGTTGTCAT
CAGTGTTTAACCGCTCGAGGatcCGGCTGCTAACAAAGCCCGAAAGGAAgC

TGAnTTGGCTGCTGCCACCGCTGAgCAATAACTAGCATAACCCCTTGGGGc
CTCTAAACGGGtCttGAGGGGTTTTTTgCTGAAaggAggAacTAtaTCCgGgAtaTc
CC

R13P

A7

>7954464.seq - ID: A7-T7 on 2011/5/23-16:15:47 automatically edited with PhredPhrap, start with base no.: 17 Internal Params: Windowsize: 20, Goodqual: 19, Badqual: 10, Minseqlength: 50, nbadelimit: 1

TnnaATaTTTTGTTtACTTtAaGAAGGAGATATACCATGGGCAGCAGCCATCatc
ctnaTCAtCACAGCAGCGGCCTGGTGCCGCGCGGCAGCCATATGGACGTTGC
TGGCTTAACTGACTGTGGTCTGATTCCCAAAAGCAATCAGGATGCTTTTTA
TATTGATGAAAAGCATCAGCGCTTTTTTATTGTTGCAGATGGCATGGGGGG
ACACGCCGGCGGCGAGGAAGCCAGTCGTTTAGCCGTCGATCACATTCGGC
AGTATTTGGAAACCCACCTTGAAGACCTGCAGCACGACCCCGTCACTCTA
CTCCGCCAAGCCTTTCTTGCAGCCAATCATGCTATTGTTGAGCAGCAGCGC
CAAAACAGTGCCCGTGCCGATATGGGTACGACGGCTGTGGTGATTCTACT
TGATGAAAAGGGCGATCGCGCTTGGTGTGCCCATGTGGGCGACTCCCGCA
TCTACCGCTGGCGCAAAGATCAACTCCAGCAGATTACCAGCGACCACACG
TGGATTGCTCAAGCCGTGCAACTGGGTAGCTTGACCATTGAGCAGGCGCG
GCAGCATCCGTGGCGCCATGTGCTCTCCCAGTGTTTAGGACGCGAGGACC
TCAGCCAAATTGATATTCAGCCCATCGACCTAGAGCCGGGCGATCGCCTG
CTGCTGTGCAGTGATGGCCTGACCGAAGAACTCACAGATGACGTCATCAG
TATCTACCTCAGTGAACCCAATGTTCAAAAAGCTGCTGCTGCCCTTGTGGA
tgCAGCCAAAACCCATGGCGGGCGTGACAATGTCACCGTCGTTGTCATCAG
TGTTTAACCGCTCGAGGATCCGGCTGCTAACAAAGCCCGAAAGGAAGCTG
AGTTGGCTGCTGCCACCGCTGAGCAATAACTAGCATAACCCCTTGGGGCC
TCTAAACGGGTcTTGAGGGGTTTTTTGCTGAAAGGagGAACTATATCCCGG
AtATCCCGCAannnGGCCCGGCAGTAccGGCATAACcaAGCCTATgCcTACAGC
ATCCAGGGTgacggTGCcnangAt

B4

>7951856.seq - ID: B4-T7 on 2011/5/21-8:50:49 automatically edited with PhredPhrap, start with base no.: 19 Internal Params: Windowsize: 20, Goodqual: 19, Badqual: 10, Minseqlength: 50, nbadelimit: 1

TnnaataTTTTGTTtACTTtAaGAAGGAGATATACCATGGGCAGCAGCCATcatC
ATCATCATCACAGCAGCGGCCTGgtGCCGCGCGGCAGCCATATGGACGtTG
CTGGCTTAACTGACTGTGGTCTGATTCCCAAAAGCAATCAGGATGCTTTTT
ATATTGATGAAAAGCATCAGCGCTTTTTTATTGTTGCAGATGGCATGGGGG
GACACGCCGGCGGCGAGGAAGCCAGTCGTTTAGCCGTCGATCACATTCGG
CAGTATTTGGAAACCCACCTTGAAGACCTGCAGCACGACCCCGTCACTCT
ACTCCGCCAAGCCTTTCTTGCAGCCAATCATGCTATTGTTGAGCAGCAGCG
CCAAAACAGTGCCCGTGCCGATATGGGTACGACGGCTGTGGTGATTCTAC
TTGATGAAAAGGGCGATCGCGCTTGGTGTGCCCATGTGGGCGACTCCCGC
ATCTACCGCTGGCGCAAAGATCAACTCCAGCAGATTACCAGCGACCACAC
GTGGATTGCTCAAGCCGTGCAACTGGGTAGCTTGACCATTGAGCAGGCGC

GGCAGCATCCGTGGCGCCATGTGCTCTCCCAGTGTTTAGGACGCGAGGAC
CTCAGCCAAATTGATATTCAGCCCATCGACCTAGAGCCGGGCGATCGCCT
GCTGCTGTGCAGTGATGGCCTGACCGAAGAACTCACAGATGACGTCATCA
GTATCTACCTCAGTGAACCCAATGTTCAAAAAGCTGCTGCTGCCCTTGTGG
ATGCAGCCAAnACCCATGGCGGGCGTGACAATGTCACCGTCGTTGTCATC
AGTGTTTAACCGCTCGAGGATCCGGCTGCTAACAAAGCCCGAAAGGAAGC
TGAGTTGGCTGCTGCCACCGCTGAGCAATAACTAGCATAACCCCTTGGgGC
CTCTAAACGGGTCTTGAGggGTTtTTTGCTGAAAGGAGGAACTATATCCGG
ATATCCCGCAAGangcccGGCAGTACcGGCATaACCAagCCTATGCCTACAGC
ATCCAGGGTGACGGTGCccaAGGAtGannaTgaa

D7

>8055292.seq - ID: D7-T7 on 2011/6/15-8:25:28 automatically edited with PhredPhrap, start with base no.: 29 Internal Params: Windowsize: 20, Goodqual: 19, Badqual: 10, Minseqlength: 50, nbadelimit: 1

ATTTTgTTTAaCTTTAAGAAGGAGAtATACCATGGGCAGCAGCCATCaTcaTC
ATCATCACAGCAGCGGCCTGGTGCCGCGCGGCAGCCATATGGACGTTGCT
GGCTTAACTGACTGTGGTCTGATTCCCAAAAGCAATCAGGATGCTTTTTAT
ATTGATGAAAAGCATCAGCGCTTTTTATTGTTGCAGATGGCATGGGGGG
ACACGCCGGCGGCGAGGAAGCCAGTCGTTTAGCCGTCGATCACATTCGGC
AGTATTTGGAAACCCACCTTGAAGACCTGCAGCACGACCCCGTCACTCTA
CTCCGCCAAGCCTTTCTTGCAGCCAATCATGCTATTGTTGAGCAGCAGCGC
CAAAACAGTGCCCGTGCCGATATGGGTACGACGGCTGTGGTGATTCTACT
TGATGAAAAGGGCGATCGCGCTTGGTGTGCCCATGTGGGCGACTCCCGCA
TCTACCGCTGGCGCAAAGATCAACTCCAGCAGATTACCAGCGACCACACG
TGGATTGCTCAAGCCGTGCAACTGGGTAGCTTGACCATTGAGCAGGCGCG
GCAGCATCCGTGGCGCCATGTGCTCTCCCAGTGTTTAGGACGCGAGGACC
TCAGCCAAATTGATATTCAGCCCATCGACCTAGAGCCGGGCGATCGCCTG
CTGCTGTGCAGTGATGGCCTGACCGAAGAACTCACAGATGACGTCATCAG
TATCTACCTCAGTGAACCCAATGTTCAAAAAGCTGCTGCTGCCCTTGTGGA
TGCAGCCAAAACCCATGGCGGGCGTGACAATGTCACCGTCGTTGTCATCA
GTGtTTAACCGCTCGAGGATcCGGCTGCTAACAAAGCCCGAAAGGAAgCTG
AnTTGncTGCTGCCaCCGCTGAgCAATAACTAgCATaAcCCCtTGGGGcCtCTA
AACGGGtCTtGnnGgTTTTTGcTGAAaggangaAcTaTaTcCGGAtatcCCGCAgAngc
CcgGcaGTACcGGc

F4

>8055305.seq - ID: F4-T7 on 2011/6/15-10:18:47 automatically edited with PhredPhrap, start with base no.: 11 Internal Params: Windowsize: 20, Goodqual: 19, Badqual: 10, Minseqlength: 50, nbadelimit: 1

aATTcCCTctnaanaTTTTGTttaCTTtAaGAAGGAGaTATACCATGGGCAGCAGCC
ATCATCATCATCATCACAGCAGCGGCCTGGTGCCGCGCGGCAGCCATATG
GACGTTGCTGGCTTAACTGACTGTGGTCTGATTCCTAAAAGCAATCAGGA
TGCTTTTTATATTGATGAAAAGCATCAGCGCTTTTTATTGTTGCAGATGG
CATGGGGGGACACGCCGGCGGCGAGGAAGCCAGTCGTTTAGCCGTCGATC
ACATTCGGCAGTATTTGGAAACCCACCTTGAAGACCTGCAGCACGACCCC

GTCACTCTACTCCGCCAAGCCTTTCTTGCAGCCAATCATGCTATTGTTGAG
CAGCAGCGCCAAAACAGTGCCCGTGCCGATATGGGTACGACGGCTGTGGT
GATTCTACTTGATGAAAAGGGCGATCGCGCTTGGTGTGCCCATGTGGGCG
ACTCCCGCATCTACCGCTGGCGCAAAGATCAACTCCAGCAGATTACCAGC
GACCACACGTGGATTGCTCAAGCCGTGCAACTGGGTAGCTTGACCATTGA
GCAGGCGCGGCAGCATCCGTGGCGCCATGTGCTCTCCCAGTGTTTAGGAC
GCGAGGACCTCAGCCAAATTGATATTCAGCCCATCGACCTAGAGCCGGGC
GATCGCCTGCTGCTGTGCAGTGATGGCCTGACCGAAGAACTCACAGATGA
CGTCATCAGTATCTACCTCAGTGAACCCAATGTTCAAAAAGCTGCTGCTGC
CCTTGTGGATGCAGCCAAnACCCATGGCGGGCGTGACAATGTCACCGTCG
TTGTCATCAGTGTTTAACCGCTCGAGGATCCGGCTGCTAACAAAGCCCGA
AAGGAAGCTGantTGGCTGCTGCCACCGCTGancAATAACTAGCATAACCCC
TTGGGGCCTCTAAACGGGTcTTGAGGgGTtTTTTGCTGAAAGGAGGAACTAT
ATCCGGannTCCCGCAAgaggcCCGGCAGTAccgGCATAACCAAGnnnaTGCcTA
CaGCATCCAGGGtgacgGT

R13Q

B2

>7951854.seq - ID: B2-T7 on 2011/5/21-8:12:44 automatically edited with PhredPhrap, start with base no.: 24 Internal Params: Windowsize: 20, Goodqual: 19, Badqual: 10, Minseqlength: 50, nbadelimit: 1
AnTTTTGTttaCTTtAaGAAGGAGATATACCATGGGCAGCAGCCaTnnncATCAT
CATCACAGCAGCGGCCTGGTGCCGCGCGGCAGCCATATGGACGTTGCTGG
CTTAACTGACTgtGGTCTGATTCAAAAAAGCAATCAGGATGCTTTTTATATT
GATGAAAAGCATCAGCGCTTTTTTATTGTTGCAGATGGCATGGGGGGACA
CGCCGGCGGCGAGGAAGCCAGTCGTTTAGCCGTCGATCACATTCGGCAGT
ATTTGGAAACCCACCTTGAAGACCTGCAGCACGACCCCGTCACTCTACTC
CGCCAAGCCTTTCTTGCAGCCAATCATGCTATTGTTGAGCAGCAGCGCCA
AAACAGTGCCCGTGCCGATATGGGTACGACGGCTGTGGTGATTCTACTTG
ATGAAAAGGGCGATCGCGCTTGGTGTGCCCATGTGGGCGACTCCCGCATC
TACCGCTGGCGCAAAGATCAACTCCAGCAGATTACCAGCGACCACACGTG
GATTGCTCAAGCCGTGCAACTGGGTAGCTTGACCATTGAGCAGGCGCGGC
AGCATCCGTGGCGCCATGTGCTCTCCCAGTGTTTAGGACGCGAGGACCTC
AGCCAAATTGATATTCAGCCCATCGACCTAGAGCCGGGCGATCGCCTGCT
GCTGTGCAGTGATGGCCTGACCGAAGAACTCACAGATGACGTCATCAGTA
TCTACCTCAGTGAACCCAATGTTCAAAAAGCTGCTGCTGCCCTTGTGGATG
CAGCCAAAACCCATGGCGGGCGTGACAATGTCACCGTCGTTGTCATCAGT
GTTTAACCGCTCGAGGATCCGGCTGCTAACAAAGCCCGAAAGGAAGCTGA
GTTGGCTGCTGCCACCGCTGAGCAATAACTAGCATAACCCCTTGGGGCCT
CTAAACGGGTCTTGAGGGGTTTTTTGCTGAAAGGAGGAACTATATCCGGA
TATCCCGCAAGAGGCCCGGCAGTACCGGCATAACCAAGCCTATGCCTACA
GCATCcagGGTGACGGTGCCGAGGATGACGATGanngCATTGTTAGaTTtCAT
ACACGgnnnCCTGACTGCgTTAGCAATTtAACTnnngataAACTACccGCATTnaaa
GCTTatc

B8

>7951859.seq - ID: B8-T7 on 2011/5/21-8:12:44 automatically edited with PhredPhrap, start with base no.: 20 Internal Params: Windowsize: 20, Goodqual: 19, Badqual: 10, Minseqlength: 50, nbadelimit: 1
aanaTTTTGTttaCTTtAaGAAGGAGATATACCATGGGCAGCAGCCaTcatcATCA
TCATCACAGCAGCGGCCTGGTGCCGCGCGGCAGCCATATGGACGTTGCTG
GCTTAACTGACTgtGGTCTGATTCAAAAAAGCAATCAGGATGCTTTTTATAT
TGATGAAAAGCATCAGCGCTTTTTTATTGTTGCAGATGGCATGGGGGGAC
ACGCCGGCGGCGAGGAAGCCAGTCGTTTAGCCGTCGATCACATTCGGCAG
TATTTGGAAACCCACCTTGAAGACCTGCAGCACGACCCCGTCACTCTACTC
CGCCAAGCCTTTCTTGCAGCCAATCATGCTATTGTTGAGCAGCAGCGCCA
AAACAGTGCCCGTGCCGATATGGGTACGACGGCTGTGGTGATTCTACTTG
ATGAAAAGGGCGATCGCGCTTGGTGTGCCCATGTGGGCGACTCCCGCATC
TACCGCTGGCGCAAAGATCAACTCCAGCAGATTACCAGCGACCACACGTG
GATTGCTCAAGCCGTGCAACTGGGTAGCTTGACCATTGAGCAGGCGCGGC
AGCATCCGTGGCGCCATGTGCTCTCCCAGTGTTTAGGACGCGAGGACCTC
AGCCAAATTGATATTCAGCCCATCGACCTAGAGCCGGGCGATCGCCTGCT
GCTGTGCAGTGATGGCCTGACCGAAGAACTCACAGATGACGTCATCAGTA
TCTACCTCAGTGAACCCAATGTTCAAAAAGCTGCTGCTGCCCTTGTGGATG
CAGCCAAAACCCATGGCGGGCGTGACAATGTCACCGTCGTTGTCATCAGT
GTTTAACCGCTCGAGGATCCGGCTGCTAACAAAGCCCGAAAGGAAGCTGA
GTTGGCTGCTGCCACCGCTGAGCAATAACTAGCATAACCCCTTGgGGCCTC
TAAACGGGTCTTGAGGGGTTTTtTGCTGAAAGGAGGAACTATATCCGGATA
TCCCGCAAGangCCCGGCAGTACCGGCATAACCAAGCCTATGCCTACAGCA
TCCAGGGTGACGGTGCCGAGGATGACGATGagcgCATTGTTagaTTtCAtACAC
GGTGCCTGACTGCgtTAGCAATTTAACTnngatnAACTACCGCATTAAaGCTTA
TcGatganaa

C5

>7951863.seq - ID: C5-T7 on 2011/5/21-8:12:39 automatically edited with PhredPhrap, start with base no.: 18 Internal Params: Windowsize: 20, Goodqual: 19, Badqual: 10, Minseqlength: 50, nbadelimit: 1
annaTTTTGTttaCTTtAaGaAGGAGATATACCATGGGCAGCAGCCATCATCATC
ATCATCACAGCAGCGGCCTgGTGCCGCGCGGCAGCCATATGGACGTTGCT
GGCTTAACTGACTGTGGTCTGATTCAGAAAAGCAATCAGGATGCTTTTTAT
ATTGATGAAAAGCATCAGCGCTTTTTTATTGTTGCAGATGGCATGGGGGG
ACACGCCGGCGGCGAGGAAGCCAGTCGTTTAGCCGTCGATCACATTCGGC
AGTATTTGGAAACCCACCTTGAAGACCTGCAGCACGACCCCGTCACTCTA
CTCCGCCAAGCCTTTCTTGCAGCCAATCATGCTATTGTTGAGCAGCAGCGC
CAAAACAGTGCCCGTGCCGATATGGGTACGACGGCTGTGGTGATTCTACT
TGATGAAAAGGGCGATCGCGCTTGGTGTGCCCATGTGGGCGACTCCCGCA
TCTACCGCTGGCGCAAAGATCAACTCCAGCAGATTACCAGCGACCACACG
TGGATTGCTCAAGCCGTGCAACTGGGTAGCTTGACCATTGAGCAGGCGCG
GCAGCATCCGTGGCGCCATGTGCTCTCCCAGTGTTTAGGACGCGAGGACC
TCAGCCAAATTGATATTCAGCCCATCGACCTAGAGCCGGGCGATCGCCTG
CTGCTGTGCAGTGATGGCCTGACCGAAGAACTCACAGATGACGTCATCAG

TATCTACCTCAGTGAACCCAATGTTCAAAAAGCTGCTGCTGCCCTTGTGGA
TGCAGCCAAAACCCATGGCGGGCGTGACAATGTCACCGTCGTTGTCATCA
GTGTTTAACCGCTCGAGGATCCGGCTGCTAACAAAGCCCGAAAGGAAGCT
GAGTTGGCTGCTGCCACCGCTGAGCAATAACTAGCATAACCCCTTGgGGC
CTCTAAACGGGTCTTGAGGGGTTTTTTGCTGAAAGGAGGAACTATATCCG
GATATCCCGCAAGAGGCCCGGCAGTACCGGCATAACCAAGCCTATGCCTA
CAGCATCCAGGGTGACGGTGCCGAGGATGACGATGAgcGCATTGTTAGAT
TtCATACACGGTGCCTGACTGCGTTAGCAATTtAACTgtgnnnAACTACcgCAT
TAAaGCTTATcGatganaagcT

R13H

A2

>7951846.seq - ID: A2-T7 on 2011/5/21-8:50:49 automatically edited with PhredPhrap, start with base no.: 24 Internal Params: Windowsize: 20, Goodqual: 19, Badqual: 10, Minseqlength: 50, nbadelimit: 1

annaTTTTGTTtACTTtAAGAAGGAGATATACCATGGGCAGCAGCCATCATCA
TCATCATCACAGCAGCGGCCTGGTGCCGCGCGGCAGCCATATGGACGTTG
CTGGCTTAACTGACTgtGGTCTGATTCATAAAAGCAATCAGGATGCTTTTTA
TATTGATGAAAAGCATCAGCGCTTTTTTATTGTTGCAGATGGCATGGGGGG
ACACGCCGGCGGCGAGGAAGCCAGTCGTTTAGCCGTCGATCACATTCGGC
AGTATTTGGAAACCCACCTTGAAGACCTGCAGCACGACCCCGTCACTCTA
CTCCGCCAAGCCTTTCTTGCAGCCAATCATGCTATTGTTGAGCAGCAGCGC
CAAAACAGTGCCCGTGCCGATATGGGTACGACGGCTGTGGTGATTCTACT
TGATGAAAAGGGCGATCGCGCTTGGTGTGCCCATGTGGGCGACTCCCGCA
TCTACCGCTGGCGCAAAGATCAACTCCAGCAGATTACCAGCGACCACACG
TGGATTGCTCAAGCCGTGCAACTGGGTAGCTTGACCATTGAGCAGGCGCG
GCAGCATCCGTGGCGCCATGTGCTCTCCCAGTGTTTAGGACGCGAGGACC
TCAGCCAAATTGATATTCAGCCCATCGACCTAGAGCCGGGCGATCGCCTG
CTGCTGTGCAGTGATGGCCTGACCGAAGAACTCACAGATGACGTCATCAG
TATCTACCTCAGTGAACCCAATGTTCAAAAAGCTGCTGCTGCCCTTGTGGA
TGCAGCCAAAACCCATGGCGGGCGTGACAATGTCACCGTCGTTGTCATCA
GTGTTTAACCGCTCGAGGATCCGGCTGCTAACAAAGCCCGAAAGGAAGCT
GAGTTGGCTGCTGCCACCGCTGAGCAATAACTAGCATAACCCCTTGGGGC
CTCTAAACGGGTCTTGAGGGGTTTTTTGCTGAAAGGAGGAACTATATCCG
GATATCCCGCAAGAGGCCCGGCAGTACCGGCATAACCAAGCCTATGCCTA
CAGCATCcAgGGTGACGGTGCCGannATGAcgaTGancGCATTGTTagaTTtCAtac
acggntgcCTGactgcgTTagcaATTtAACT

D4

>8055289.seq - ID: D4-T7 on 2011/6/15-10:18:47 automatically edited with PhredPhrap, start with base no.: 11 Internal Params: Windowsize: 20, Goodqual: 19, Badqual: 10, Minseqlength: 50, nbadelimit: 1

aATTCCcTCtannaaATTTtGTTtaACTTtAAGAAGGAGATATACCATGGgCAGCA
GCCATCATCATCATCATCACAGCAGCGGCCTGGTGCCGCGCGGCAGCCAT
ATGGACGTTGCTGGCTTAACTGACTGTGGTCTGATtcaCAAAAGCAATCAGG
ATGCTTTTTATATTGATGAAAAGCATCAGCGCTTTTTTATTGTTGCAGATG

GCATGGGGGACACGCCGGCGGCGAGGAAGCCAGTCGTTTAGCCGTCGA
TCACATTCGGCAGTATTTGGAAACCCACCTTGAAGACCTGCAGCACGACC
CCGTCACTCTACTCCGCCAAGCCTTTCTTGCAGCCAATCATGCTATTGTTG
AGCAGCAGCGCCAAAACAGTGCCCGTGCCGATATGGGTACGACGGCTGTG
GTGATTCTACTTGATGAAAAGGGCGATCGCGCTTGGTGTGCCCATGTGGG
CGACTCCCGCATCTACCGCTGGCGCAAAGATCAACTCCAGCAGATTACCA
GCGACCACACGTGGATTGCTCAAGCCGTGCAACTGGGTAGCTTGACCATT
GAGCAGGCGCGGCAGCATCCGTGGCGCCATGTGCTCTCCCAGTGTTTAGG
ACGCGAGGACCTCAGCCAAATTGATATTCAGCCCATCGACCTAGaGCCGG
GCGATCgccTGCTGCTGTGCagtGATGGCCTGACCgaaGAACTCACAgATGACG
TCATcagTATCTACccnnngTGAACcccaATGTtccaAnanncTgcnGCtnnccccttgggGa

E5

>8055298.seq - ID: E5-T7 on 2011/6/15-10:18:47 automatically edited with PhredPhrap, start with base no.: 19 Internal Params: Windowsize: 20, Goodqual: 19, Badqual: 10, Minseqlength: 50, nbadelimit: 1

aannTaATTTtGTTtaACTTtAAGAAGGAGaTATACCATGGGCAGCAGCCATCA
TCATCATCATCACAGCAGCGGCCTGGTGCCGCGCGGCAGCCATATGGACG
TTGCTGGCTTAACTGACTGTGGTCTGATTCACAAAAGCAATCAGGATGCTT
TTTATATTGATGAAAAGCATCAGCGCTTTTTTATTGTTGCAGATGGCATGG
GGGGACACGCCGGCGGCGAGGAAGCCAGTCGTTTAGCCGTCGATCACATT
CGGCAGTATTTGGAAACCCACCTTGAAGACCTGCAGCACGACCCCGTCAC
TCTACTCCGCCAAGCCTTTCTTGCAGCCAATCATGCTATTGTTGAGCAGCA
GCGCCAAAACAGTGCCCGTGCCGATATGGGTACGACGGCTGTGGTGATTC
TACTTGATGAAAAGGGCGATCGCGCTTGGTGTGCCCATGTGGGCGACTCC
CGCATCTACCGCTGGCGCAAAGATCAACTCCAGCAGATTACCAGCGACCA
CACGTGGATTGCTCAAGCCGTGCAACTGGGTAGCTTGACCATTGAGCAGG
CGCGGCAGCATCCGTGGCGCCATGTGCTCTCCCAGTGTTTAGGACGCGAG
GaCCTCAGCCAAATTGATATTCAGCCCATCGACCTAGAGCCGGGCGATCG
CCTGCTGCTGTGCAGTGATGgcnTGACCGAAGAACTCACAGATGACGTCAT
CAGTATCTACcttcaGTGAACcCCAATGTTCAAAAAAGCTnncTGCttgCcntTGTG
GGATGCAGCncnnaanCCCATGgnnGGGGCgttgACAattGTCAcccGTCGTtTGTCA
TcaagtgtTTTAACccGcttCGangGATcCCGGcttGCtnaACAAAaGCCCCGAAAAGG
aaacctgaaatttGGC

R13G

A3

>7951847.seq - ID: A3-T7 on 2011/5/21-8:12:39 automatically edited with PhredPhrap, start with base no.: 10 Internal Params: Windowsize: 20, Goodqual: 19, Badqual: 10, Minseqlength: 50, nbadelimit: 1

CatTCCcTcTnnaataTTTTGTttaCTTtAaGAAGGAGaTATACCATGGGCAGCAGC
CATcnncATCATCATCACAGCAGCGGCCTGgtGCCGCGCGGCAGCCATATGG
ACGTTGCTGGCTTAACTGACTgtGGTCTGATTGGAAAAAGCAATCAGGATG
CTTTTTATATTGATGAAAAGCATCAGCGCTTTTTTATTGTTGCAGATGGCA
TGGGGGGACACGCCGGCGGCGAGGAAGCCAGTCGTTTAGCCGTCGATCAC
ATTCGGCAGTATTTGGAAACCCACCTTGAAGACCTGCAGCACGACCCCGT

CACTCTACTCCGCCAAGCCTTTCTTGCAGCCAATCATGCTATTGTTGAGCA
GCAGCGCCAAAACAGTGCCCGTGCCGATATGGGTACGACGGCTGTGGTGA
TTCTACTTGATGAAAAGGGCGATCGCGCTTGGTGTGCCCATGTGGGCGAC
TCCCGCATCTACCGCTGGCGCAAAGATCAACTCCAGCAGATTACCAGCGA
CCACACGTgGATTGCTCAAGCCGTGCAACTGGGTAGCTTGACCATTGAGC
AGGCGCGGCAGCATCCGTGGCGCCATGTGCTCTCCCAGTGTTTAGGACGC
GAGGACCTCAGCCAAATTGATATTCAGCCCATCGACCTAGAGCCGGGCGA
TCGCCTGCTGCTGTGCAGTGATGGCCTGACCGAAGAACTCACAGATGACG
TCATCAGTATCTACCTCAGTGAACCCAATGTTCAAAAAGCTGCTGCTGCCC
TTGTGGATGCAGCCAAAACCCATGGCGGGCGTGACAATGTCACCGTCGTT
GTCATCAGTGTTTAACCGCTCGAGGATCCGGCTGCTAACAAAGCCCGAAA
GGAAGCTGAGTTGGCTGCTGCCACCGCTGAGCAATAACTAGCATAACCCC
TTGgGGCCTCTAAACGGGTCTTGAGGGGTTTTTTGCTGAAAGGaggAACTAT
ATCCGGATATCCCGCAAGaggCCCGGCAGTACCGGCATAACCAAGCCTATG
CCTACAGCATCCAGgGTGACGGTGCcnaGGATGACgaTGAGCgCATTGTTAg
ATTtCAtAcacGgtgCCTGACTGCGTTAGCAATTTAACTGtgnnnAACTACcGCAT
TAAAgCTTatcgat

A4

>7951848.seq - ID: A4-T7 on 2011/5/21-8:50:45 automatically edited with PhredPhrap, start with base no.: 9 Internal Params: Windowsize: 20, Goodqual: 19, Badqual: 10, Minseqlength: 50, nbadelimit: 1

aATTCCcTcnanaataTTTTGTTtACTTtAaGAAGGAGATATACCATGGGCAGCAG
CCATCATCATCATCATCACAGCAGCGGCCTGgtGCCGCGCGGCAGCCATAT
GGACGTTGCTGGCTTAACTGACTgtGGTCTGATTGGGAAAAGCAATCAGGA
TGCTTTTTATATTGATGAAAAGCATCAGCGCTTTTTTATTGTTGCAGATGG
CATGGGGGGACACGCCGGCGGCGAGGAAGCCAGTCGTTTAGCCGTCGATC
ACATTCGGCAGTATTTGGAAACCCACCTTGAAGACCTGCAGCACGACCCC
GTCACTCTACTCCGCCAAGCCTTTCTTGCAGCCAATCATGCTATTGTTGAG
CAGCAGCGCCAAAACAGTGCCCGTGCCGATATGGGTACGACGGCTGTGGT
GATTCTACTTGATGAAAAGGGCGATCGCGCTTGGTGTGCCCATGTGGGCG
ACTCCCGCATCTACCGCTGGCGCAAAGATCAACTCCAGCAGATTACCAGC
GACCACACGTGGATTGCTCAAGCCGTGCAACTGGGTAGCTTGACCATTGA
GCAGGCGCGGCAGCATCCGTGGCGCCATGTGCTCTCCCAGTGTTTAGGAC
GCGAGGACCTCAGCCAAATTGATATTCAGCCCATCGACCTAGAGCCGGGC
GATCGCCTGCTGCTGTGCAGTGATGGCCTGACCGAAGAACTCACAGATGA
CGTCATCAGTATCTACCTCAGTGAACCCAATGTTCAAAAAGCTGCTGCTGC
CCTTGTGGATGCAGCCAAnACCCATGGCGGGCGTGACAATGTCACCGTCG
TTGTCATCAGTGTTTAACCGCTCGAGGATCCGGCTGCTAACAAAGCCCGA
AAGGAAGCTGAGTTGGCTGCTGCCACCGCTGAGCAATAACTAGCATAACC
CCTTGGGGCCTCTAAACGGGTCTTGAGGGGTTTTTTGCTGAAAGGAGGAA
CTATATCCGGATATCCCGCAAGAGGCCCGGCAGTACCGGCATAACCAAGC
CTATGCCTACAGCATCCAGGGTGACGGTGCCgAGGATGACgaTgaacGCATT
GTTagaTTtCATACaCGGtgCCTGACTGCGTTAGCAATTtAactgnnnAtaAAcTACC
GCATTAAAgCTTATcGaTgntnagct

R13N
A8
>7951852.seq - ID: A8-T7 on 2011/5/21-8:12:44 automatically edited with PhredPhrap, start with base no.: 20 Internal Params: Windowsize: 20, Goodqual: 19, Badqual: 10, Minseqlength: 50, nbadelimit: 1
annAaTTTTGTTtACTTtAaGAAGGAGATATACCATGGGCAGCAGCCATcnncA
TCATCATCACAGCAGCGGCCTgGTGCCGCGCGGCAGCCATATGGACGTTG
CTGGCTTAACTGACTgtGGTCTGATTAACAAAAGCAATCAGGATGCTTTTT
ATATTGATGAAAAGCATCAGCGCTTTTTTATTGTTGCAGATGGCATGGGGG
GACACGCCGGCGGCGAGGAAGCCAGTCGTTTAGCCGTCGATCACATTCGG
CAGTATTTGGAAACCCACCTTGAAGACCTGCAGCACGACCCCGTCACTCT
ACTCCGCCAAGCCTTTCTTGCAGCCAATCATGCTATTGTTGAGCAGCAGCG
CCAAAACAGTGCCCGTGCCGATATGGGTACGACGGCTGTGGTGATTCTAC
TTGATGAAAAGGGCGATCGCGCTTGGTGTGCCCATGTGGGCGACTCCCGC
ATCTACCGCTGGCGCAAAGATCAACTCCAGCAGATTACCAGCGACCACAC
GTGGATTGCTCAAGCCGTGCAACTGGGTAGCTTGACCATTGAGCAGGCGC
GGCAGCATCCGTGGCGCCATGTGCTCTCCCAGTGTTTAGGACGCGAGGAC
CTCAGCCAAATTGATATTCAGCCCATCGACCTAGAGCCGGGCGATCGCCT
GCTGCTGTGCAGTGATGGCCTGACCGAAGAACTCACAGATGACGTCATCA
GTATCTACCTCAGTGAACCCAATGTTCAAAAAGCTGCTGCTGCCCTTGTGG
ATGCAGCCAAAACCCATGGCGGGCGTGACAATGTCACCGTCGTTGTCATC
AGTGTTTAACCGCTCGAGGATCCGGCTGCTAACAAAGCCCGAAAGGAAGC
TGAGTTGGCTGCTGCCACCGCTGAGCAATAACTAGCATAACCCCTTGGgGC
CTCTAAACGGGTCTTGAGGGGTTTTTTGCTGAAAGGAGGAACTATATCCG
GATATCCCGCAAGAGGCCCGGCAGTACCGGCATAACCAAGCCTATGCCTA
CAGCATCCAGGGTGACGGTGCcgaGGAtgACGATGAGcGCATTGTTAgaTTTC
ATacacGGTGCCTGACTGCGTTAGCAATTtAACTGTGATAAACTAccGCATTA
AAGCTTatcGaTganaagct
C7
>7951865.seq - ID: C7-T7 on 2011/5/21-8:12:39 automatically edited with PhredPhrap, start with base no.: 20 Internal Params: Windowsize: 20, Goodqual: 19, Badqual: 10, Minseqlength: 50, nbadelimit: 1
annaTTTTGTttaCTTtAaGAAGGAGATATACCATGGGCAGCAGCCaTcnncATCA
TCATCACAGCAGCGGCCTGGTGCCGCGCGGCAGCCATATGGACGtTGCTG
GCTTAACTGACTGTGGTCTGATTAATAAAAGCAATCAGGATGCTTTTTATA
TTGATGAAAAGCATCAGCGCTTTTTTATTGTTGCAGATGGCATGGGGGGA
CACGCCGGCGGCGAGGAAGCCAGTCGTTTAGCCGTCGATCACATTCGGCA
GTATTTGGAAACCCACCTTGAAGACCTGCAGCACGACCCCGTCACTCTAC
TCCGCCAAGCCTTTCTTGCAGCCAATCATGCTATTGTTGAGCAGCAGCGCC
AAAACAGTGCCCGTGCCGATATGGGTACGACGGCTGTGGTGATTCTACTT
GATGAAAAGGGCGATCGCGCTTGGTGTGCCCATGTGGGCGACTCCCGCAT
CTACCGCTGGCGCAAAGATCAACTCCAGCAGATTACCAGCGACCACACGT
GGATTGCTCAAGCCGTGCAACTGGGTAGCTTGACCATTGAGCAGGCGCGG
CAGCATCCGTGGCGCCATGTGCTCTCCCAGTGTTTAGGACGCGAGGACCT

CAGCCAAATTGATATTCAGCCCATCGACCTAGAGCCGGGCGATCGCCTGC
TGCTGTGCAGTGATGGCCTGACCGAAGAACTCACAGATGACGTCATCAGT
ATCTACCTCAGTGAACCCAATGTTCAAAAAGCTGCTGCTGCCCTTGTGGAT
GCAGCCAAAACCCATGGCGGGCGTGACAATGTCACCGTCGTTGTCATCAG
TGTTTAACCGCTCGAGGATCCGGCTGCTAACAAAGCCCGAAAGGAAGCTG
AGTTGGCTGCTGCCACCGCTGAGCAATAACTAGCATAACCCCTTGgGGCCT
CTAAACGGGTCTTGAGGGGTTTTTTGCTGAAAGGAGGAACTATATCCGGA
TATCCCGCAAGAGGCCCGGCAGTACCGGCATAACCAAGCCTATGCCTACA
GCATCCAGGGTGAcgGTGCcgaGGATGACGATGAGCGCATTGTTAgaTTTCAT
AcacggnngCCTGACTGCGTTAGCAATTtAACTgtgataAACTACCGCATTAAAGc
TTATcGATgnnaagcT

R13S

B5

>7951857.seq - ID: B5-T7 on 2011/5/21-8:12:44 automatically edited with PhredPhrap, start with base no.: 10 Internal Params: Windowsize: 20, Goodqual: 19, Badqual: 10, Minseqlength: 50, nbadelimit: 1

aTTCCcTcTnnaataTTTTGTTtaCTTtAaGAAGGAGATATACCATGGGCAGCAGC
CATnnncATCATCATCACAGCAGCGGCCTGGTGCCGCGCGGCAGCCATATG
GACGTTGCTGGCTTAACTGACTGTGGTCTGATTTCGAAAAGCAATCAGGA
TGCTTTTTATATTGATGAAAAGCATCAGCGCTTTTTTATTGTTGCAGATGG
CATGGGGGGACACGCCGGCGGCGAGGAAGCCAGTCGTTTAGCCGTCGATC
ACATTCGGCAGTATTTGGAAACCCACCTTGAAGACCTGCAGCACGACCCC
GTCACTCTACTCCGCCAAGCCTTTCTTGCAGCCAATCATGCTATTGTTGAG
CAGCAGCGCCAAAACAGTGCCCGTGCCGATATGGGTACGACGGCTGTGGT
GATTCTACTTGATGAAAAGGGCGATCGCGCTTGGTGTGCCCATGTGGGCG
ACTCCCGCATCTACCGCTGGCGCAAAGATCAACTCCAGCAGATTACCAGC
GACCACACGTGGATTGCTCAAGCCGTGCAACTGGGTAGCTTGACCATTGA
GCAGGCGCGGCAGCATCCGTGGCGCCATGTGCTCTCCCAGTGTTTAGGAC
GCGAGGACCTCAGCCAAATTGATATTCAGCCCATCGACCTAGAGCCGGGC
GATCGCCTGCTGCTGTGCAGTGATGGCCTGACCGAAGAACTCACAGATGA
CGTCATCAGTATCTACCTCAGTGAACCCAATGTTCAAAAAGCTGCTGCTGC
CCTTGTGGATGCAGCCAAAACCCATGGCGGGCGTGACAATGTCACCGTCG
TTGTCATCAGTGTTTAACCGCTCGAGGATCCGGCTGCTAACAAAGCCCGAn
AGGAAGCTGAGTTGGCTGCTGCCACCGCTGAGCAATAACTAGCATAACCC
CTTGgGGCCTCTAAACGGGTCTTGAGGGGTTTTTTGCTGAAAGGAGGAACT
ATATCCGGATATCCCGCAAGAGGCCCGGCAGTACCGGCATAACCAAGCCT
ATGCCTACAGCATCCAGGGTGACGGTGCCGAGGATGACGaTGancgCATTGT
TAGATTtCATACACGGTgCCTGACTGCGTTAGCAATTTAACTgtgatnAACTAC
CGCATTAAaGCTTATcGATgataa

D6

>8055291.seq - ID: D6-T7 on 2011/6/15-10:18:43 automatically edited with PhredPhrap, start with base no.: 22 Internal Params: Windowsize: 20, Goodqual: 19, Badqual: 10, Minseqlength: 50, nbadelimit: 1

annaTTTtGTTTACTTtAaGAAGGAGATATACCATGGGCAGCAGCCATCATCA

TCATCATCACAGCAGCGGCCTGGTGCCGCGCGGCAGCCATATGGACGTTG
CTGGCTTAACTGACTGTGGTCTGATTTCAAAAAGCAATCAGGATGCTTTTT
ATATTGATGAAAAGCATCAGCGCTTTTTTATTGTTGCAGATGGCATGGGGG
GACACGCCGGCGGCGAGGAAGCCAGTCGTTTAGCCGTCGATCACATTCGG
CAGTATTTGGAAACCCACCTTGAAGACCTGCAGCACGACCCCGTCACTCT
ACTCCGCCAAGCCTTTCTTGCAGCCAATCATGCTATTGTTGAGCAGCAGCG
CCAAAACAGTGCCCGTGCCGATATGGGTACGACGGCTGTGGTGATTCTAC
TTGATGAAAAGGGCGATCGCGCTTGGTGTGCCCATGTGGGCGACTCCCGC
ATCTACCGCTGGCGCAAAGATCAACTCCAGCAGATTACCAGCGACCACAC
GTGGATTGCTCAAGCCGTGCAACTGGGTAGCTTGACCATTGAGCAGGCGC
GGCAGCATCCGTGGCGCCATGTGCTCTCCCAGTGTTTAGGACGCGAGGAC
CTCAGCCAAATTGATATTCAGCCCATCGACCTAGAGCCGGGCGATCGCCT
GCTGCTGTGCAGTGATGGCCTGACCGAAGAACTCACAGATGACGTCATCA
GTATCTACCTCAGTGAACCCAATGTTCAAAAAGCTGCTGCTGCCCTTGTGG
ATGCAGCCAAnACCCATGGCGGGCGTGACAATGTCACCGTCGTTGTCATC
AGTGTTTAACCGCTCGAGGATCCGGCTGCTAACAAAGCCCGAAAGGAAGC
TGAGTTGGCTGCTGCCACCGCTGancAATAACTAGCATAACCCCTtngGGGC
CTCTAAancGGGTcTTGAGggngTTTTTGCTGAAAGGaggAACTATATcnngGat
ATCCCGCAanaaGGCccgGCAGTACCgGCATAACCAanCctAngccTACAGCATC
CaagGGTgangg

R13T

C1

>7951860.seq - ID: C1-T7 on 2011/5/21-8:12:39 automatically edited with PhredPhrap, start with base no.: 22 Internal Params: Windowsize: 20, Goodqual: 19, Badqual: 10, Minseqlength: 50, nbadelimit: 1

annaTTTtGTTtACTTtAaGAAGGAGaTATACCATGGGCAGCAGCCATCaTCATC
ATCATCACAGCAGCGGCCTGgtGCCGCGCGGCAGCCATATGGACGTTGCTG
GCTTAACTGACTGTGGTCTGATTACAAAAAGCAATCAGGATGCTTTTTATA
TTGATGAAAAGCATCAGCGCTTTTTTATTGTTGCAGATGGCATGGGGGGA
CACGCCGGCGGCGAGGAAGCCAGTCGTTTAGCCGTCGATCACATTCGGCA
GTATTTGGAAACCCACCTTGAAGACCTGCAGCACGACCCCGTCACTCTAC
TCCGCCAAGCCTTTCTTGCAGCCAATCATGCTATTGTTGAGCAGCAGCGCC
AAAACAGTGCCCGTGCCGATATGGGTACGACGGCTGTGGTGATTCTACTT
GATGAAAAGGGCGATCGCGCTTGGTGTGCCCATGTGGGCGACTCCCGCAT
CTACCGCTGGCGCAAAGATCAACTCCAGCAGATTACCAGCGACCACACGT
GGATTGCTCAAGCCGTGCAACTGGGTAGCTTGACCATTGAGCAGGCGCGG
CAGCATCCGTGGCGCCATGTGCTCTCCCAGTGTTTAGGACGCGAGGACCT
CAGCCAAATTGATATTCAGCCCATCGACCTAGAGCCGGGCGATCGCCTGC
TGCTGTGCAGTGATGGCCTGACCGAAGAACTCACAGATGACGTCATCAGT
ATCTACCTCAGTGAACCCAATGTTCAAAAAGCTGCTGCTGCCCTTGTGGAT
GCAGCCAAAACCCATGGCGGGCGTGACAATGTCACCGTCGTTGTCATCAG
TGTTTAACCGCTCGAGGATCCGGCTGCTAACAAAGCCCGAAAGGAAGCTG
AGTTGGCTGCTGCCACCGCTGAGCAATAACTAGCATAACCCCTTGGGGCC
TCTAAACGGGTCTTGAGGGGTTTTTTGCTGAAAGGAGGAACTATATCCGG

ATATCCCGCAAGAGGCCCGGCAGTACCGGCATAACCAAGCCTATGCCTAC
AGCATCCAGGGTGACGGTGCcgaGGATGACGATGAGCGCATTgttAgATTtCA
TACaCGGTGCCTGACTGCGTTAGCAATTtAACTnngatnAACTACcGCATTAAa
GCTTATcGATGanaa

D8

>8055293.seq - ID: D8-T7 on 2011/6/15-10:18:47 automatically edited with PhredPhrap, start with base no.: 21 Internal Params: Windowsize: 20, Goodqual: 19, Badqual: 10, Minseqlength: 50, nbadelimit: 1

anaaATTTtGTTtAaCTTtAAGAAGGAGATATACCATGGGCAGCAGCCATCATC
ATCATCATCACAGCAGCGGCCTGGTGCCGCGCGGCAGCCATATGGACGTT
GCTGGCTTAACTGACTGTGGTCTGATTAcTAAAAGCAATCAGGATGCCAAT
GTTCAAAAAGCTGCTGCTGCCCTTGTGGATGCAGCCAAAACCCATGGCGG
GCGTGACAATGTCACCGTCGTTGTCATCAGTGTTTAACCGCTCGAGGATCC
GGCTGCTAACAAAGCCCGAAAGGAAGCTGAGTTGGCTGCTGCCACCGCTG
AGCAATAACTAGCATAACCCCTTGGGGCCTCTAAACGGGTCTTGAGGGGT
TTTTTGCTGAAAGGAGGAACTATATCCGGATATCCCGCAAGAGGCCCGGC
AGTACCGGCATAACCAAGCCTATGCCTACAGCATCCAGGGTGACGGTGCC
GAGGATGACGATGAGCGCATTGTTAGATTTCATACACGGTGCCTGACTGC
GTTAGCAATTTAACTGTGATAAACTACCGCATTAAAGCTTATCGATGATAA
GCTGTCAAACATGAGAATTCTTGAAGACGAAAGGGCCTCGTGATACGCCT
ATTTTTATAGGTTAATGTCATGATAATAATGGTTTCTTAGACGTCAGGTGG
CACTTTTCGGGGAAATGTGCGCGGAACCCCTATTTGTTTATTTTTCTAAAT
ACATTCAAATATGTATCCGCTCATGAGACAATAACCCTGATAAATGCTTC
AATAATATTGAAnAAGGAAGAGTATGAGTATTCAACATTTCCGTGTCGCC
CTTATTCcCTTTTTTGCGGCATTTTGCCTTCCTGTTTTTGCTCACcCAGAAAC
GCTGGTGAAAGTAAAAgATGCTGAAGATCAGTTGgGTGCACGAGTGGGTT
ACATCGAACTGGATCTCAACAGCGGTAAgaTCCTTGagantTTTcncCCCGAana
ACGTTTTCCAATGATGaGcACTTTTAAAGTTCTGCTAtGtGGcgcGGtnTTATCC
cgtgTTGannccGGGCAanannAACTnnggTCgccgCa

E8

>8055301.seq - ID: E8-T7 on 2011/6/15-8:25:30 automatically edited with PhredPhrap, start with base no.: 31 Internal Params: Windowsize: 20, Goodqual: 19, Badqual: 10, Minseqlength: 50, nbadelimit: 1

tTTTGTTTAaCTTTAAGAAGGAGAtATACCATGGGCAGCAGCCATCatcaTCAT
CATCACAGCAGCGGCCTGGTGCCGCGCGGCAGCCATATGGACGTTGCTGG
CTTAACTGACTGTGGTCTGATTACCAAAAGCAATCAGGATGCTTTTTATAT
TGATGAAAAGCATCAGCGCTTTTTTATTGTTGCAGATGGCATGGGGGGAC
ACGCCGGCGGCGAGGAAGCCAGTCGTTTAGCCGTCGATCACATTCGGCAG
TATTTGGAAACCCACCTTGAAGACCTGCAGCACGACCCCGTCACTCTACTC
CGCCAAGCCTTTCTTGCAGCCAATCATGCTATTGTTGAGCAGCAGCGCCA
AAACAGTGCCCGTGCCGATATGGGTACGACGGCTGTGGTGATTCTACTTG
ATGAAAAGGGCGATCGCGCTTGGTGTGCCCATGTGGGCGACTCCCGCATC
TACCGCTGGCGCAAAGATCAACTCCAGCAGATTACCAGCGACCACACGTG
GATTGCTCAAGCCGTGCAACTGGGTAGCTTGACCATTGAGCAGGCGCGGC

AGCATCCGTGGCGCCATGTGCTCTCCCAGTGTTTAGGACGCGAGGACCTC
AGCCAAATTGATATTCAGCCCATCGACCTAGAGCCGGGCGATCGCCTGCT
GCTGTGCAGTGATGGCCTGACCGAAGAACTCACAGATGACGTCATCAGTA
TCTACCTCAGTGAACCCAATGtTCAAAAAGCTGCTGCTGCCCTTGTGGAtGC
AGCCnAAACCCATGGCGGGCGTGACaATGTCACCGTCGTTGTCATCaGTGT
TTAACCGCTCGAgGATCCGGCTGCTaACAAAgcCCGAAAGGAAgCTGAnTTG
gCTGCTGcCACCGCTGAnCAATAaCTAgCATAACCCCtTGGGGncTCTAAACG
GGTCTtGAgGGGtTTTTTgcTGAAagnangaact

R13D

C6

>7951864.seq - ID: C6-T7 on 2011/5/21-8:12:44 automatically edited with PhredPhrap, start with base no.: 20 Internal Params: Windowsize: 20, Goodqual: 19, Badqual: 10, Minseqlength: 50, nbadelimit: 1

annaTTTtGTTtaCTTtAaGAAGGAGATATACCATGGGCAGCAGCCATCATCAT
CATCATCACAGCAGCGGCCTGGTGCCGCGCGGCAGCCATATGGACGTTGC
TGGCTTAACTGACTGTGGTCTGATTGATAAAAGCAATCAGGATGCTTTTTA
TATTGATGAAAAGCATCAGCGCTTTTTTATTGTTGCAGATGGCATGGGGGG
ACACGCCGGCGGCGAGGAAGCCAGTCGTTTAGCCGTCGATCACATTCGGC
AGTATTTGGAAACCCACCTTGAAGACCTGCAGCACGACCCCGTCACTCTA
CTCCGCCAAGCCTTTCTTGCAGCCAATCATGCTATTGTTGAGCAGCAGCGC
CAAAACAGTGCCCGTGCCGATATGGGTACGACGGCTGTGGTGATTCTACT
TGATGAAAAGGGCGATCGCGCTTGGTGTGCCCATGTGGGCGACTCCCGCA
TCTACCGCTGGCGCAAAGATCAACTCCAGCAGATTACCAGCGACCACACG
TGGATTGCTCAAGCCGTGCAACTGGGTAGCTTGACCATTGAGCAGGCGCG
GCAGCATCCGTGGCGCCATGTGCTCTCCCAGTGTTTAGGACGCGAGGACC
TCAGCCAAATTGATATTCAGCCCATCGACCTAGAGCCGGGCGATCGCCTG
CTGCTGTGCAGTGATGGCCTGACCGAAGAACTCACAGATGACGTCATCAG
TATCTACCTCAGTGAACCCAATGTTCAAAAAGCTGCTGCTGCCCTTGTGGA
TGCAGCCAAAACCCATGGCGGGCGTGACAATGTCACCGTCGTTGTCATCA
GTGTTTAACCGCTCGAGGATCCGGCTGCTAACAAAGCCCGAAAGGAAGCT
GAGTTGGCTGCTGCCACCGCTGAGCAATAACTAGCATAACCcCTTGGGGC
CTCTAAACGGGTCTTGAGGGGTTTTTTGCTGAAAGGAGGAACTATATCCG
GATATCCCGCAAGAGGCCCGGCAGTACCGGCATAACCAAGCCTATGCCTA
CAGCATCCAGGGTGACGGTGCCGAGGATGACGATGancGCATTGTTAgaTTT
CATACACGGTGCCTGACTGCGTTAGCAATTTAACTgngannAACTACCGCAT
TAAAGCTTATcGATGanaagcTGtcAAa

E4

>8055297.seq - ID: E4-T7 on 2011/6/15-8:25:30 automatically edited with PhredPhrap, start with base no.: 23 Internal Params: Windowsize: 20, Goodqual: 19, Badqual: 10, Minseqlength: 50, nbadelimit: 1

AgaAatAatTTTgtTTAACTTTAAGAAGGAGAtATACCATGGGCAGCAGCCATC
atcaTCATCATCACAGCAGCGGCCTGGTGCCGCGCGGCAGCCATATGGACGT
TGCTGGCTTAACTGACTGTGGTCTGATTGACAAAAGCAATCAGGATGCTTT
TTATATTGATGAAAAGCATCAGCGCTTTTTTATTGTTGCAGATGGCATGGG

GGGACACGCCGGCGGCGAGGAAGCCAGTCGTTTAGCCGTCGATCACATTC
GGCAGTATTTGGAAACCCACCTTGAAGACCTGCAGCACGACCCCGTCACT
CTACTCCGCCAAGCCTTTCTTGCAGCCAATCATGCTATTGTTGAGCAGCAG
CGCCAAAACAGTGCCCGTGCCGATATGGGTACGACGGCTGTGGTGATTCT
ACTTGATGAAAAGGGCGATCGCGCTTGGTGTGCCCATGTGGGCGACTCCC
GCATCTACCGCTGGCGCAAAGATCAACTCCAGCAGATTACCAGCGACCAC
ACGTGGATTGCTCAAGCCGTGCAACTGGGTAGCTTGACCATTGAGCAGGC
GCGGCAGCATCCGTGGCGCCATGTGCTCTCCCAGTGTTTAGGACGCGAGG
ACCTCAGCCAAATTGATATTCAGCCCATCGACCTAGAGCCGGGCGATCGC
CTGCTGCTGTGCAGTGATGGCCTGACCGAAGAACTCACAGATGACGTCAT
CAGTATCTACCTCAGTGAACCCAATGtTCaAAAAGCTGCTGCTGCCCTTGTG
GAtGCAGCCAAAACCCATGGCGGGCGTGACAATGTCACCGTCGTTGTCATC
AGtGttTaACCGCTCGAGGATcCGGCTGCTaACAAAGcCCGAAAGGAAgCTGA
gTngGCTGCTGCCaCCGCTGAgCaaTaACTAgCATAACCCcnngGGGcCtCTAAaC
GgnnntngAGGGTTTTTGCTgaAaggAngaa

F7

>8055308.seq - ID: F7-T7 on 2011/6/15-10:18:43 automatically edited with PhredPhrap, start with base no.: 9 Internal Params: Windowsize: 20, Goodqual: 19, Badqual: 10, Minseqlength: 50, nbadelimit: 1

aaTTCCCTCTagaaTaATTTTGTTtAaCTTtAaGAAGgagaTATACCATGGGCAGCA
GCCATCATCATCATCATCACAGCAGCGGCCTggtGCCGCGCGGCAGCCATA
TGGACGTTGCTGGCTTAACTGACTgtgGTCTGATTGCAAAAAGCAATCAGgA
TGCTTTTTATATTGATGAAAAGCATCAGCGCTTTTTTATTGTTGCAGATGG
CATGGGGGGACACGCCGGCGGCGAGGaAGCCAGTCGTTTAGCCGTCGATC
ACATTCGGCAGTATTTGGAAACCCACCtTGAAGACCTGCAGCACGACCCCG
TCACTCTACTCCGCCAAGCCTTTCTTGCAGCCAATCATGCTATTGTTGAGC
AGCAGCGCCAAAACAGTGCCCGTGCCGATATGGGTACGACGGCTGTGGTG
ATTCTACTTGATGAAAAGGGCGATCGCGCTTGGTGTGCCCAtgTGGGCGAC
TCCCGCATCTACCGCTGGCGCAAAGATCAACTCCAGCAGATTACCAGCGA
CCACACGTGGATTGCTCAAGCCGTGCAACTGGGTAGCTTGACCATTGAGC
AGGCGCGGCAGCATCCGTGGCGCCATGTGCTCTCCCAGTGTTTAGGACGC
GAGGACCTCAGCCAAAttngATATTCAgccCCAtcCGACCtaagaagnccgGGCGAT
CgncCTGCtgccTGTGCAgttGATGggcctggACCGAAagAACtcnaCAgaatGAcngtCAt
ccanTATcttacct

R13F

B6

>7951858.seq - ID: B6-T7 on 2011/5/21-8:12:39 automatically edited with PhredPhrap, start with base no.: 23 Internal Params: Windowsize: 20, Goodqual: 19, Badqual: 10, Minseqlength: 50, nbadelimit: 1

AnTTTTGTttaCTTtAaGAAGGAGATATACCATGGGCAGCAGCCATCATcATC
ATCATCACAGCAGCGGCCTGgtGCCGCGCGGCAGCCATATGGACGTTGCTG
GCTTAACTGACTGTGGTCTGATTTTTAAAAGCAATCAGGATGCTTTTTATA
TTGATGAAAAGCATCAGCGCTTTTTTATTGTTGCAGATGGCATGGGGGGA
CACGCCGGCGGCGAGGAAGCCAGTCGTTTAGCCGTCGATCACATTCGGCA

GTATTTGGAAACCCACCTTGAAGACCTGCAGCACGACCCCGTCACTCTAC
TCCGCCAAGCCTTTCTTGCAGCCAATCATGCTATTGTTGAGCAGCAGCGCC
AAAACAGTGCCCGTGCCGATATGGGTACGACGGCTGTGGTGATTCTACTT
GATGAAAAGGGCGATCGCGCTTGGTGTGCCCATGTGGGCGACTCCCGCAT
CTACCGCTGGCGCAAAGATCAACTCCAGCAGATTACCAGCGACCACACGT
GGATTGCTCAAGCCGTGCAACTGGGTAGCTTGACCATTGAGCAGGCGCGG
CAGCATCCGTGGCGCCATGTGCTCTCCCAGTGTTTAGGACGCGAGGACCT
CAGCCAAATTGATATTCAGCCCATCGACCTAGAGCCGGGCGATCGCCTGC
TGCTGTGCAGTGATGGCCTGACCGAAGAACTCACAGATGACGTCATCAGT
ATCTACCTCAGTGAACCCAATGTTCAAAAAGCTGCTGCTGCCCTTGTGGAT
GCAGCCAAAACCCATGGCGGGCGTGACAATGTCACCGTCGTTGTCATCAG
TGTTTAACCGCTCGAGGATCCGGCTGCTAACAAAGCCCGAAAGGAAGCTG
AGTTGGCTGCTGCCACCGCTGAGCAATAACTAGCATAACCCCTTGGGGCC
TCTAAACGGGTCTTGAGGGGTTTTTTGCTGAAAGGAGGAACTATATCCGG
ATATCCCGCAAGaggCCCGGCAGTACCGGCATAACCAAGCCTATGCCTACA
GCATCCAGGGTGACGGTGCCGAGGATGACGATGAgcgCATTGTTAgaTTtCA
TacacGGTGCCTGACTGCGTTAGCAATTtAACTGTGatnAACTACCGCATTAAA
GCTTATcGatgnnaanntgtcAAaCAtnngAattCTTGAa

E1

>8055294.seq - ID: E1-T7 on 2011/6/15-8:25:29 automatically edited with PhredPhrap, start with base no.: 30 Internal Params: Windowsize: 20, Goodqual: 19, Badqual: 10, Minseqlength: 50, nbadelimit: 1
TTTTgtTTAaCTTTAAGAAGGAGAtATACCATGGGCAGCAGCCATCntcatcATC
ATCACAGCAGCGGCCTGGTGCCGCGCGGCAGCCATATGGACGTTGCTGGC
TTAACTGACTGTGGTCTGATTTTCAAAAGCAATCAGGATGCTTTTTATATT
GATGAAAAGCATCAGCGCTTTTTTATTGTTGCAGATGGCATGGGGGGACA
CGCCGGCGGCGAGGAAGCCAGTCGTTTAGCCGTCGATCACATTCGGCAGT
ATTTGGAAACCCACCTTGAAGACCTGCAGCACGACCCCGTCACTCTACTC
CGCCAAGCCTTTCTTGCAGCCAATCATGCTATTGTTGAGCAGCAGCGCCA
AAACAGTGCCCGTGCCGATATGGGTACGACGGCTGTGGTGATTCTACTTG
ATGAAAAGGGCGATCGCGCTTGGTGTGCCCATGTGGGCGACTCCCGCATC
TACCGCTGGCGCAAAGATCAACTCCAGCAGATTACCAGCGACCACACGTG
GATTGCTCAAGCCGTGCAACTGGGTAGCTTGACCATTGAGCAGGCGCGGC
AGCATCCGTGGCGCCATGTGCTCTCCCAGTGTTTAGGACGCGAGGACCTC
AGCCAAATTGATATTCAGCCCATCGACCTAGAGCCGGGCGATCGCCTGCT
GCTGTGCAGTGATGGCCTGACCGAAGAACTCACAGATGACGTCATCAGTA
TCTACCTCAGTGAacCCAATGTTCaAAAAGCTGCTGCTGcCCTtGTGGATGCA
GCCaAAAcCCATGGCGGGCGTGACaATGTCACCGTCGTTGTCATCAGTGTTT
aACCGCTCGAGGatcCGGCTGCTAACAAAGCCCGAAAGGAAgCTGAnTTGgC
TGCTGcCACCGCtGAnCAaTAACTAGCATAACCCCttGGGGCCtCTAAacnGGtC
ttGAGGGGTTTTTTGCTGannGAggaacTATa

R13W

A5

>7951849.seq - ID: A5-T7 on 2011/5/21-8:50:49 automatically edited with

PhredPhrap, start with base no.: 24 Internal Params: Windowsize: 20, Goodqual: 19, Badqual: 10, Minseqlength: 50, nbadelimit: 1
AnTTTTGTttaCTTtAaGAAGGAGaTATACCATGGGCAGCAGCCATcatCATCAT
CATCACAGCAGCGGCCTgGTGCCGCGCGGCAGCCATATGGACGTTGCTGG
CTTAACTGACTGTGGTCTGATTTGGAAAAGCAATCAGGATGCTTTTTATAT
TGATGAAAAGCATCAGCGCTTTTTATTGTTGCAGATGGCATGGGGGGAC
ACGCCGGCGGCGAGGAAGCCAGTCGTTTAGCCGTCGATCACATTCGGCAG
TATTTGGAAACCCACCTTGAAGACCTGCAGCACGACCCCGTCACTCTACTC
CGCCAAGCCTTTCTTGCAGCCAATCATGCTATTGTTGAGCAGCAGCGCCA
AAACAGTGCCCGTGCCGATATGGGTACGACGGCTGTGGTGATTCTACTTG
ATGAAAAGGGCGATCGCGCTTGGTGTGCCCATGTGGGCGACTCCCGCATC
TACCGCTGGCGCAAAGATCAACTCCAGCAGATTACCAGCGACCACACGTG
GATTGCTCAAGCCGTGCAACTGGGTAGCTTGACCATTGAGCAGGCGCGGC
AGCATCCGTGGCGCCATGTGCTCTCCCAGTGTTTAGGACGCGAGGACCTC
AGCCAAATTGATATTCAGCCCATCGACCTAGAGCCGGGCGATCGCCTGCT
GCTGTGCAGTGATGGCCTGACCGAAGAACTCACAGATGACGTCATCAGTA
TCTACCTCAGTGAACCCAATGTTCAAAAAGCTGCTGCTGCCCTTGTGGATG
CAGCCAAAACCCATGGCGGGCGTGACAATGTCACCGTCGTTGTCATCAGT
GTTTAACCGCTCGAGGATCCGGCTGCTAACAAAGCCCGAAAGGAAGCTGA
GTTGGCTGCTGCCACCGCTGagcAATAACTAGCATAACCCCTTGGGGCCTCT
AAACgGGTCTTGAGGGGTTTTTTGCTGAAAGGAGGAACTATATccgGATATC
CCGCAagAGGCCCGGCAGTACCGGCATAACcAAGCCTATGCCTACAGCATC
CaaGGGTGAcGGTGCcnaaGGatGAcgaTGa

R13I

A1

>7951845.seq - ID: A1-T7 on 2011/5/21-8:50:45 automatically edited with PhredPhrap, start with base no.: 11 Internal Params: Windowsize: 20, Goodqual: 19, Badqual: 10, Minseqlength: 50, nbadelimit: 1
aaTTCCcTcTnnaataTTTtGTttaCTTtAAGAAGGAGATATACCATGGGCAGCAGC
CATCATCATCATCATCACAGCAGCGGCCTgGTGCCGCGCGGCAGCCATAT
GGACGTTGCTGGCTTAACTGACTgtGGTCTGATTATTAAAAGCAATCAGGA
TGCTTTTTATATTGATGAAAAGCATCAGCGCTTTTTATTGTTGCAGATGG
CATGGGGGGACACGCCGGCGGCGAGGAAGCCAGTCGTTTAGCCGTCGATC
ACATTCGGCAGTATTTGGAAACCCACCTTGAAGACCTGCAGCACGACCCC
GTCACTCTACTCCGCCAAGCCTTTCTTGCAGCCAATCATGCTATTGTTGAG
CAGCAGCGCCAAAACAGTGCCCGTGCCGATATGGGTACGACGGCTGTGGT
GATTCTACTTGATGAAAAGGGCGATCGCGCTTGGTGTGCCCATGTGGGCG
ACTCCCGCATCTACCGCTGGCGCAAAGATCAACTCCAGCAGATTACCAGC
GACCACACGTGGATTGCTCAAGCCGTGCAACTGGGTAGCTTGACCATTGA
GCAGGCGCGGCAGCATCCGTGGCGCCATGTGCTCTCCCAGTGTTTAGGAC
GCGAGGACCTCAGCCAAATTGATATTCAGCCCATCGACCTAGAGCCGGGC
GATCGCCTGCTGCTGTGCAGTGATGGCCTGACCGAAGAACTCACAGATGA
CGTCATCAGTATCTACCTCAGTGAACCCAATGTTCAAAAAGCTGCTGCTGC
CCTTGTGGATGCAGCCAAAACCCATGGCGGGCGTGACAATGTCACCGTCG

TTGTCATCAGTGTTTAACCGCTCGAGGATCCGGCTGCTAACAAAGCCCGA
AAGGAAGCTGAGTTGGCTGCTGCCACCGCTGAGCAATAACTAGCATAACC
CCTTGgGGCCTCTAAACGGGTCTTGAGGGGTTTTTTGCTGAAAGGAGGAAC
TATATCCGGAtATCCCGCAAGAGGCCCGGCAGTACCGGCATAACCAAGCC
TATGCCTACAGCATCCAGGGTGACGGTGCcgaggATGacgATgaacgcATTGTTag
aTTtCatAcacggnngCCTGACTGCGTTAGcAATTtAACTGggnnnAACTACcgcaTTA
AagCTTnncGa

R13K

C4

>7951862.seq - ID: C4-T7 on 2011/5/21-8:12:44 automatically edited with PhredPhrap, start with base no.: 22 Internal Params: Windowsize: 20, Goodqual: 19, Badqual: 10, Minseqlength: 50, nbadelimit: 1

AaTTTtGTttaCTTtAaGAAGGAGATATACCATGGGCAGCAGCCATCATCATCA
TCATCACAGCAGCGGCCTGGTGCCGCGCGGCAGCCATATGGACGTTGCTG
GCTTAACTGACTGTGGTCTGATTAAAAAAAGCAATCAGGATGCTTTTTATA
TTGATGAAAAGCATCAGCGCTTTTTTATTGTTGCAGATGGCATGGGGGGA
CACGCCGGCGGCGAGGAAGCCAGTCGTTTAGCCGTCGATCACATTCGGCA
GTATTTGGAAACCCACCTTGAAGACCTGCAGCACGACCCCGTCACTCTAC
TCCGCCAAGCCTTTCTTGCAGCCAATCATGCTATTGTTGAGCAGCAGCGCC
AAAACAGTGCCCGTGCCGATATGGGTACGACGGCTGTGGTGATTCTACTT
GATGAAAAGGGCGATCGCGCTTGGTGTGCCCATGTGGGCGACTCCCGCAT
CTACCGCTGGCGCAAAGATCAACTCCAGCAGATTACCAGCGACCACACGT
GGATTGCTCAAGCCGTGCAACTGGGTAGCTTGACCATTGAGCAGGCGCGG
CAGCATCCGTGGCGCCATGTGCTCTCCCAGTGTTTAGGACGCGAGGACCT
CAGCCAAATTGATATTCAGCCCATCGACCTAGAGCCGGGCGATCGCCTGC
TGCTGTGCAGTGATGGCCTGACCGAAGAACTCACAGATGACGTCATCAGT
ATCTACCTCAGTGAACCCAATGTTCAAAAAGCTGCTGCTGCCCTTGTGGAT
GCAGCCAAAACCCATGGCGGGCGTGACAATGTCACCGTCGTTGTCATCAG
TGTTTAACCGCTCGAGGATCCGGCTGCTAACAAAGCCCGAAAGGAAGCTG
AGTTGGCTGCTGCCACCGCTGAGCAATAACTAGCATAACCcCTTGGgGCCT
CTAAACGGGTCTTGAGGGGTTTTTTGCTGAAAGGAGGAACTATATCCGGA
TATCCCGCAAGAGGCCCGGCAGTACCGGCATAACCAAGCCTATGCCTACA
GCATCCAGgGTGACGGTGCCGaggATGACGATgaacGCATTGTTAGATTtCAT
ACACGgTGCCTGACTGCGTTAGCAATTtAACTnngatnAACTAccGCATTAAaG
CTTATcgaTGntaagcTgtCAA

R13M

D3

>8055288.seq - ID: D3-T7 on 2011/6/15-10:18:43 automatically edited with PhredPhrap, start with base no.: 11 Internal Params: Windowsize: 20, Goodqual: 19, Badqual: 10, Minseqlength: 50, nbadelimit: 1

aaTTCCcTcnnaaaaaTTTTGTttaCTTtAAGAAGGAGaTATACCATGGGCAGCAGC
CaTCATCATCATCATCACAGCAGCGGCCTGGTGCCGCGCGGCAGCCATATG
GACGTTGCTGGCTTAACTGACTGTGGTCTGATTATGAAAAGCAATCAGGA
TGCTTTTTATATTGATGAAAAGCATCAGCGCTTTTTTATTGTTGCAGATGG

CATGGGGGACACGCCGGCGGCGAGGAAGCCAGTCGTTTAGCCGTCGATC
ACATTCGGCAGTATTTGGAAACCCACCTTGAAGACCTGCAGCACGACCCC
GTCACTCTACTCCGCCAAGCCTTTCTTGCAGCCAATCATGCTATTGTTGAG
CAGCAGCGCCAAAACAGTGCCCGTGCCGATATGGGTACGACGGCTGTGGT
GATTCTACTTGATGAAAAGGGCGATCGCGCTTGGTGTGCCCATGTGGGCG
ACTCCCGCATCTACCGCTGGCGCAAAGATCAACTCCAGCAGATTACCAGC
GACCACACGTGGATTGCTCAAGCCGTGCAACTGGGTAGCTTGACCATTGA
GCAGGCGCGGCAGCATCCGTGGCGCCATGTGCTCTCCCAGTGTTTAGGAC
GCGAGGACCTCAGCCAAATTGATATTCAGCCCATCGACCTAGAGCCGGGC
GATCGCCTGCTGCTGTGCAGTGATGGCCTGACCGAAGAACTCACAGATGA
CGTCATCAGTATCTACCTCAGTGAACCCAATGTTCAAAAAGCTGCTGCTGC
CCTTGTGGATGCAGCCAAnACcCATGGCGGgCGTGACAATGTCACCGTCGT
TGTCATCAGTGTTTAACCGCTCGAGGATcCGGCTGCTAACAAAGCCCGAA
AGGAAGCTGagtTgGCTGCTGCCACcncTGAGCAatancTAGCanaacCCcttngggCC
TCTAancGgg

R13Y

F2

>8055303.seq - ID: F2-T7 on 2011/6/15-8:25:25 automatically edited with PhredPhrap, start with base no.: 22 Internal Params: Windowsize: 20, Goodqual: 19, Badqual: 10, Minseqlength: 50, nbadelimit: 1
CtAgAntAAtTTTgTTTAaCTTTAAGAAGGAGAtATACCATGGGCAGCAGCCAT
CATCATCATCATCACAGCAGCGGCCTGGTGCCGCGCGGCAGCCATATGGA
CGTTGCTGGCTTAACTGACTGTGGTCTGATTTATAAAAGCAATCAGGATGC
TTTTTATATTGATGAAAAGCATCAGCGCTTTTTTATTGTTGCAGATGGCAT
GGGGGACACGCCGGCGGCGAGGAAGCCAGTCGTTTAGCCGTCGATCAC
ATTCGGCAGTATTTGGAAACCCACCTTGAAGACCTGCAGCACGACCCCGT
CACTCTACTCCGCCAAGCCTTTCTTGCAGCCAATCATGCTATTGTTGAGCA
GCAGCGCCAAAACAGTGCCCGTGCCGATATGGGTACGACGGCTGTGGTGA
TTCTACTTGATGAAAAGGGCGATCGCGCTTGGTGTGCCCATGTGGGCGAC
TCCCGCATCTACCGCTGGCGCAAAGATCAACTCCAGCAGATTACCAGCGA
CCACACGTGGATTGCTCAAGCCGTGCAACTGGGTAGCTTGACCATTGAGC
AGGCGCGGCAGCATCCGTGGCGCCATGTGCTCTCCCAGTGTTTAGGACGC
GAGGACCTCAGCCAAATTGATATTCAGCCCATCGACCTAGAGCCGGGCGA
TCGCCTGCTGCTGTGCAGTGATGGCCTGACCGAAGAACTCACAGATGACG
TCATCAGTATCTACCTCAGTGAACCCAATGTTCAAAAAGCTGCTGCTGCCC
TTGTGGATGCAGCCAAAACCCATGGCGGGCGTGACAATGTCACCGTCGTT
GTCATCAGTGTTTAACCGCTCGAGGaTCCGGCTGCTAACAAAGCCCGAAAg
GAAgCTGAnTtgGCTGCTGCCACCGCTGAgCAatAACtAGCATAaCCCCttGGGG
cCTCTAAACGGGtCtTGAgGGGTTTTtnctGAAAggAggaaCTatATCCgGanaTCCC
gCa

Unknown variants

B3

>7951855.seq - ID: B3-T7 on 2011/5/21-8:12:39 automatically edited with PhredPhrap, start with base no.: 23 Internal Params: Windowsize: 20, Goodqual: 19,

Badqual: 10, Minseqlength: 50, nbadelimit: 1
AnTTTTGTTtACTTtAaGAAGGAGATATACCATGGGCAGCAGCCATcatcATCA
TCATCACAGCAGCGGCCTGgtGCCGCGCGGCAGCCATATGGACGTTGCTGG
CTTAACTGACTGTGGTCTGATtnaTAAAAGCAATCAGGATGCTTTTTATATT
GATGAAAAGCATCAGCGCTTTTTTATTGTTGCAGATGGCATGGGGGGACA
CGCCGGCGGCGAGGAAGCCAGTCGTTTAGCCGTCGATCACATTCGGCAGT
ATTTGGAAACCCACCTTGAAGACCTGCAGCACGACCCCGTCACTCTACTC
CGCCAAGCCTTTCTTGCAGCCAATCATGCTATTGTTGAGCAGCAGCGCCA
AAACAGTGCCCGTGCCGATATGGGTACGACGGCTGTGGTGATTCTACTTG
ATGAAAAGGGCGATCGCGCTTGGTGTGCCCATGTGGGCGACTCCCGCATC
TACCGCTGGCGCAAAGATCAACTCCAGCAGATTACCAGCGACCACACGTG
GATTGCTCAAGCCGTGCAACTGGGTAGCTTGACCATTGAGCAGGCGCGGC
AGCATCCGTGGCGCCATGTGCTCTCCCAGTGTTTAGGACGCGAGGACCTC
AGCCAAATTGATATTCAGCCCATCGACCTAGAGCCGGGCGATCGCCTGCT
GCTGTGCAGTGATGGCCTGACCGAAGAACTCACAGATGACGTCATCAGTA
TCTACCTCAGTGAACCCAATGTTCAAAAAGCTGCTGCTGCCCTTGTGGATG
CAGCCAAAACCCATGGCGGGCGTGACAATGTCACCGTCGTTGTCATCAGT
GTTTAACCGCTCGAGGATCCGGCTGCTAACAAAGCCCGAAAGGAAGCTGA
GTTGGCTGCTGCCACCGCTGAGCAATAACTAGCATAACCCCTTGgGGCCTC
TAAACGGGTCTTGAgGgGTTTTTTGCTGAAAGGAGGAACTATATCCGGATA
TCCCGCAAGAGGCCCGGCAGTACCGGCATAACCAAGCCTATGCCTACAGC
ATCCAGGGTGACGGTGCCgaGGATGAcGATGAGCgCATTGTTAGATTtCATA
CacGGTGCCTgacTGcgTTAgcAATTtAACTnnngatnAACTACcgcATTAAAGCTTa
tcGatganaa

D1

>8055286.seq - ID: D1-T7 on 2011/6/15-8:25:27 automatically edited with PhredPhrap, start with base no.: 28 Internal Params: Windowsize: 20, Goodqual: 19, Badqual: 10, Minseqlength: 50, nbadelimit: 1
ATTTTgTTTAACTTTAAGAAGGAGAtATACCATGGGCAGCAGCCATCaTcaTC
ATCATCACAGCAGCGGCCTGGTGCCGCGCGGCAGCCATATGGACGTTGCT
GGCTTAACTGACTGTGGTCTGATTnnnaaaAGCAATCAGGATGCTTTTTATAT
TGATGAAAAGCATCAGCGCTTTTTTATTGTTGCAGATGGCATGGGGGGAC
ACGCCGGCGGCGAGGAAGCCAGTCGTTTAGCCGTCGATCACATTCGGCAG
TATTTGGAAACCCACCTTGAAGACCTGCAGCACGACCCCGTCACTCTACTC
CGCCAAGCCTTTCTTGCAGCCAATCATGCTATTGTTGAGCAGCAGCGCCA
AAACAGTGCCCGTGCCGATATGGGTACGACGGCTGTGGTGATTCTACTTG
ATGAAAAGGGCGATCGCGCTTGGTGTGCCCATGTGGGCGACTCCCGCATC
TACCGCTGGCGCAAAGATCAACTCCAGCAGATTACCAGCGACCACACGTG
GATTGCTCAAGCCGTGCAACTGGGTAGCTTGACCATTGAGCAGGCGCGGC
AGCATCCGTGGCGCCATGTGCTCTCCCAGTGTTTAGGACGCGAGGACCTC
AGCCAAATTGATATTCAGCCCATCGACCTAGAGCCGGGCGATCGCCTGCT
GCTGTGCAGTGATGGCCTGACCGAAGAACTCACAGATGACGTCATCAGTA
TCTACCTCAGTGAACCCAATGTTCAAAAAGCTGCTGCTGcCCTTGTGGATG
CAGCCAAAACCCATGGCGGGCGTGACAATGTCACCGTCGTTGTCaTCAGT

GTTtAACCGCTCGAGGAtCCGGCTGCTaACAAAGCCCGAAAGGAAGCTGAg
TTGGCTGCTGCCACCGCTGAnCAATAACTAGCATAACCCCtTGGGGcCtCTA
AACGGGtCTTGAGGGgtTTTTTGCTGAAaggAggaacTATATCCGgAt

E2

>8055295.seq - ID: E2-T7 on 2011/6/15-8:25:30 automatically edited with PhredPhrap, start with base no.: 30 Internal Params: Windowsize: 20, Goodqual: 19, Badqual: 10, Minseqlength: 50, nbadelimit: 1

ATTTTGtTTaACTTTAAGAAGGAGAtATACCATGGGCAGCAGCCATCatCaTC
ATCATCACAGCAGCGGCCTGGTGCCGCGCGGCAGCCATATGGACGTTGCT
GGCTTAACTGACTGTGGTCTGATTcTnanAAGCAATCAGGATGCTTTTTATA
TTGATGAAAAGCATCAGCGCTTTTTTATTGTTGCAGATGGCATGGGGGGA
CACGCCGGCGGCGAGGAAGCCAGTCGTTTAGCCGTCGATCACATTCGGCA
GTATTTGGAAACCCACCTTGAAGACCTGCAGCACGACCCCGTCACTCTAC
TCCGCCAAGCCTTTCTTGCAGCCAATCATGCTATTGTTGAGCAGCAGCGCC
AAAACAGTGCCCGTGCCGATATGGGTACGACGGCTGTGGTGATTCTACTT
GATGAAAAGGGCGATCGCGCTTGGTGTGCCCATGTGGGCGACTCCCGCAT
CTACCGCTGGCGCAAAGATCAACTCCAGCAGATTACCAGCGACCACACGT
GGATTGCTCAAGCCGTGCAACTGGGTAGCTTGACCATTGAGCAGGCGCGG
CAGCATCCGTGGCGCCATGTGCTCTCCCAGTGTTTAGGACGCGAGGACCT
CAGCCAAATTGATATTCAGCCCATCGACCTAGAGCCGGGCGATCGCCTGC
TGCTGTGCAGTGATGGCCTGACCGAAGAACTCACAGATGACGTCATCAGT
ATCTACCTCAGTGAACCCAATGtTCAAAAAGCTGCTGCTGCCCTTGTGGAT
GCAGCCaAAACCCATGGCGGGCGTGACAATGTCACCGTCGTTGTCATCAG
TGTTTaACCGCTCGAgGATCCGGCTGCtnACAAAGcCCGAAAGGAAGCTGAg
TTGgcTGCTGcCaCCGCTGAgCAATAACTAgCAtAaCCCcTtgGGGCCTCTAAAc
gGGTCtTGAGGGGtTTTTTtGcTgAAAGGAgGAAcTAtaTCCgG

F6

>8055307.seq - ID: F6-T7 on 2011/6/15-10:18:47 automatically edited with PhredPhrap, start with base no.: 28 Internal Params: Windowsize: 20, Goodqual: 19, Badqual: 10, Minseqlength: 50, nbadelimit: 1

ATTTtGTTtaACTtnAAGaAGgagaTATACCAtggGGCAGCAGCCATCATCATCAT
CATCACAGCAGCGGCCTGGTGCCGCGCGGCAGCCATATGGACGTTGCTGG
CTTAACTGACTGTgGTCTGATTcngAAAAGCAATCAGGATGCTTTTTATATT
GATGAAAAGCATCAGCGCTTTTTTATTGTTGCAGATGGCATGGGGGGACA
CGCCGGCGGCGAgGAAGCCAGTCGTTTAGCCGTCGATCACATTCGGCAGT
ATTTGGAAACCCACCTTGAAGACCTGCAGCACGACCCCGTCACTCTACTC
CGCCAAGCCTTTCTTGCAGCCAATCATGCTATTGTTGAGCAGCAGCGCCA
AAACAGTGCCCGTGCCGATATGGGTACGACGGCTGTGGTGATTCTACTTG
ATGAAAAGGGCGATCGCGCTTGGTGTGCCCATGTGGGCGACTCCCGCATC
TACCGCTGGCGCAAAGATCAACTCCAGCAGATTACCAGCGACCACACGTG
GATTGCTCAAGCCGTGCAACTgnnTAGCTTGACCATTGAGcagGCGCGgcaGC
ATcCgTGGCGCCATgtgCTCTCCcnntGTTTAGGACGCGaGGACCCTCAgccaaAT
TGATATTtCAGCCCAtcgAcctaGagcCggGcg

Supplementary file 2:

The generated tPphA variants from random site-directed mutagenesis and the gene codes of variants amino acids replacing wild type tPphA Arg-13.

The gene code of Arg-13 of Wild type tPphA is CAC. The plasmids extracted from *E.coli* X-10 Gold colony B7, C2, D2, D5 did not yield any results in DNA sequencing.

Variants name	The gene codes of variants amino acids replacing wild type tPphA Arg-13	The name of *E.coli* X-10 Gold colony
R13L	CCT	A6
	CTC	C3
	TTA	E6
	CTT	E7
	CTC	F1
	TTA	F5
	CTT	F8
R13R	CAC	B1
	CGT	C8
	CAC	E3
	CGT	F3
R13P	CCC	A7
	CCC	B4
	CCC	D7
	CCT	F4
R13Q	CAA	B2
	CAA	B8
	CAG	C5
R13H	CAT	A2
	CAC	D4
	CAC	E5
R13G	GGA	A3
	GGG	A4
R13N	AAC	A8
	AAT	C7
R13S	TCG	B5
	TCA	D6
R13T	ACA	C1
	ACT	D8
	ACC	E8
R13D	GAT	C6
	GAC	E4
	GAC	F7
R13F	TTT	B6
	TTC	E1
R13W	TGG	A5
R13I	ATT	A1

R13K	AAA	C4
R13M	ATG	D3
R13Y	TAT	F2
Unknown variants	N*AT	B3
	NNN	D1
	CTN	E2
	CNG	F6

N* means four nucleotides AGCT could randomly appear at that position

Supplementary file 3:

SDS-PAGE of wild type tPphAand R13 variants

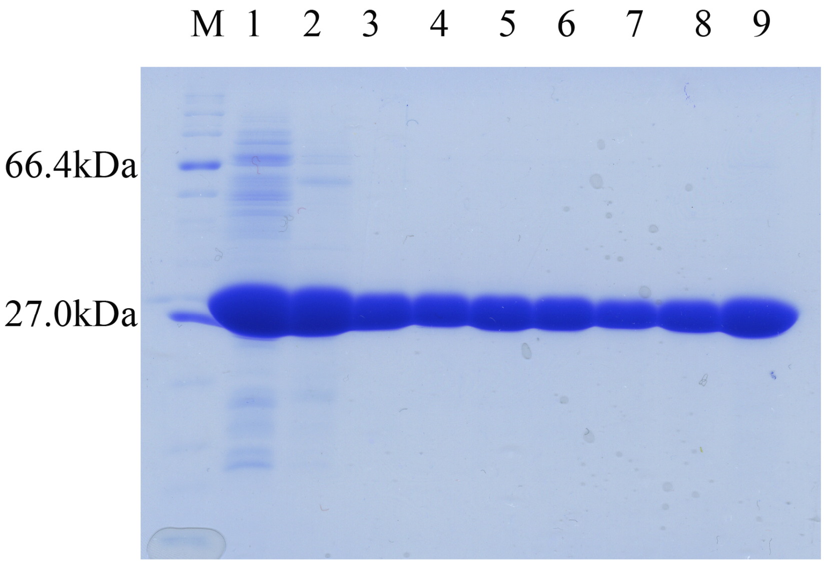

M: ProteinMarker
1: wild type tPphA
2: R13K
3: R13H
4: R13D
5: R13S
6: R13T
7: R13N
8: R13Q
9: R13A

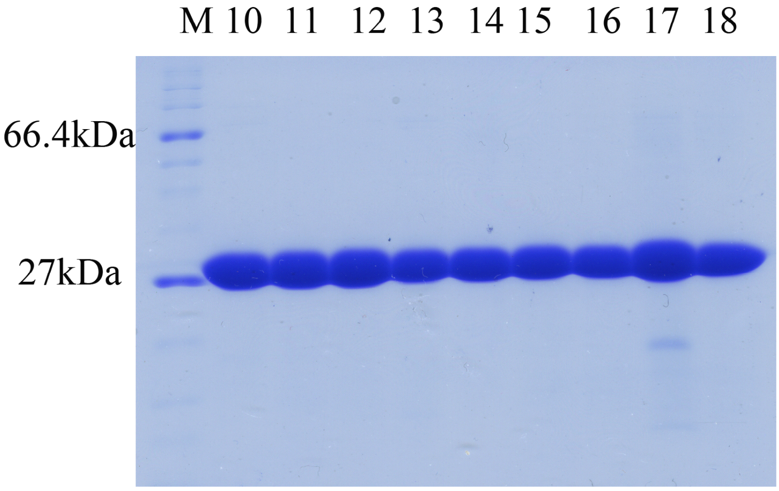

M: ProteinMarker
10: R13L
11: R13I
12: R13M
13: R13F
14: R13W
15: R13Y
16: R13G
17: R13C
18: R13P
